# Intra- and Inter-individual Differences in the Human Intestinal Microbial Conversion of (-)-Epicatechin and Bioactivity of Its Major Colonic Metabolite 5-(3′,4′-Dihydroxy-Phenyl)-γ-Valerolactone in Regulating Nrf2-Mediated Gene Expression

**DOI:** 10.3389/fnut.2022.910785

**Published:** 2022-06-30

**Authors:** Chen Liu, Sjef Boeren, Ivonne M. C. M. Rietjens

**Affiliations:** ^1^Division of Toxicology, Wageningen University and Research, Wageningen, Netherlands; ^2^Laboratory of Biochemistry, Wageningen University and Research, Wageningen, Netherlands

**Keywords:** (-)-Epicatechin, inter-individual difference, intra-individual difference, microbial conversion, Nrf2, 5-(3′, 4′-dihydroxyphenyl)-γ-valerolactone

## Abstract

(-)-Epicatechin (EC) is one of the most popular polyphenols present in various food products in daily life. Upon intake, it is intensively metabolized by microbiota in the large intestine. In the present study, intra- and inter-individual variations in this gut microbial conversion of EC and the concomitant formation of its major metabolites, including 5-(3′,4′-dihydroxy phenyl)-γ-valerolactone (3,4-diHPV), were identified and quantified *via* liquid chromatography triple quadrupole mass spectrometry (LC-TQ-MS) in anaerobic fecal incubations. In addition, the bioactivity of EC and 3,4-diHPV in activating Nrf2-mediated gene expression was tested quantifying their effects in the U2OS Nrf2 CALUX assay (a reporter gene assay that is used to test the potency of chemicals in activation of Nrf2 signaling), and on the expression levels of Nrf2-related proteins in Hepa1c1c7 and Caco-2 cells *via* nanoLC-MSMS. A quantitative real-time polymerase chain reaction (RT-qPCR) was carried out to confirm selected Nrf2-regulated gene expressions at the mRNA level. Results obtained show that both intra- and inter-individual differences exist in human gut microbial EC degradation and 3,4-diHPV formation, with inter-individual differences being more distinct than intra-individual differences. The metabolite, 3,4-diHPV, showed higher potency in the U2OS Nrf2 CALUX assay than EC itself. Among the obviously altered Nrf2-related proteins, 14 and 10 Nrf2-associated proteins were upregulated to a higher extent upon 3,4-diHPV treatment than in the EC treated group for Hepa1c1c7 and Caco-2 cells, respectively. While only three and four of these Nrf2-associated proteins were induced at a higher level upon EC than upon 3,4-diHPV treatment for Hepa1c1c7 and Caco-2 cells, respectively. RT-qPCR results showed that indeed Nrf2-mediated genes (e.g., Nqo1 and Ugt1a) were only induced significantly in 3,4-diHPV treated and not in EC treated Hepa1c1c7 cells. Taken together, the results suggest that the major colonic EC metabolite, 3,4-diHPV, was more capable of inducing Nrf2-mediated gene expression than its parent compound EC. This implies that the evident inter- and intra-individual differences in the microbial conversion of EC to this major metabolite 3,4-diHPV may affect the overall health-promoting effects of EC consumption related to the Nrf2 pathway activation.

**Graphical Abstract:**
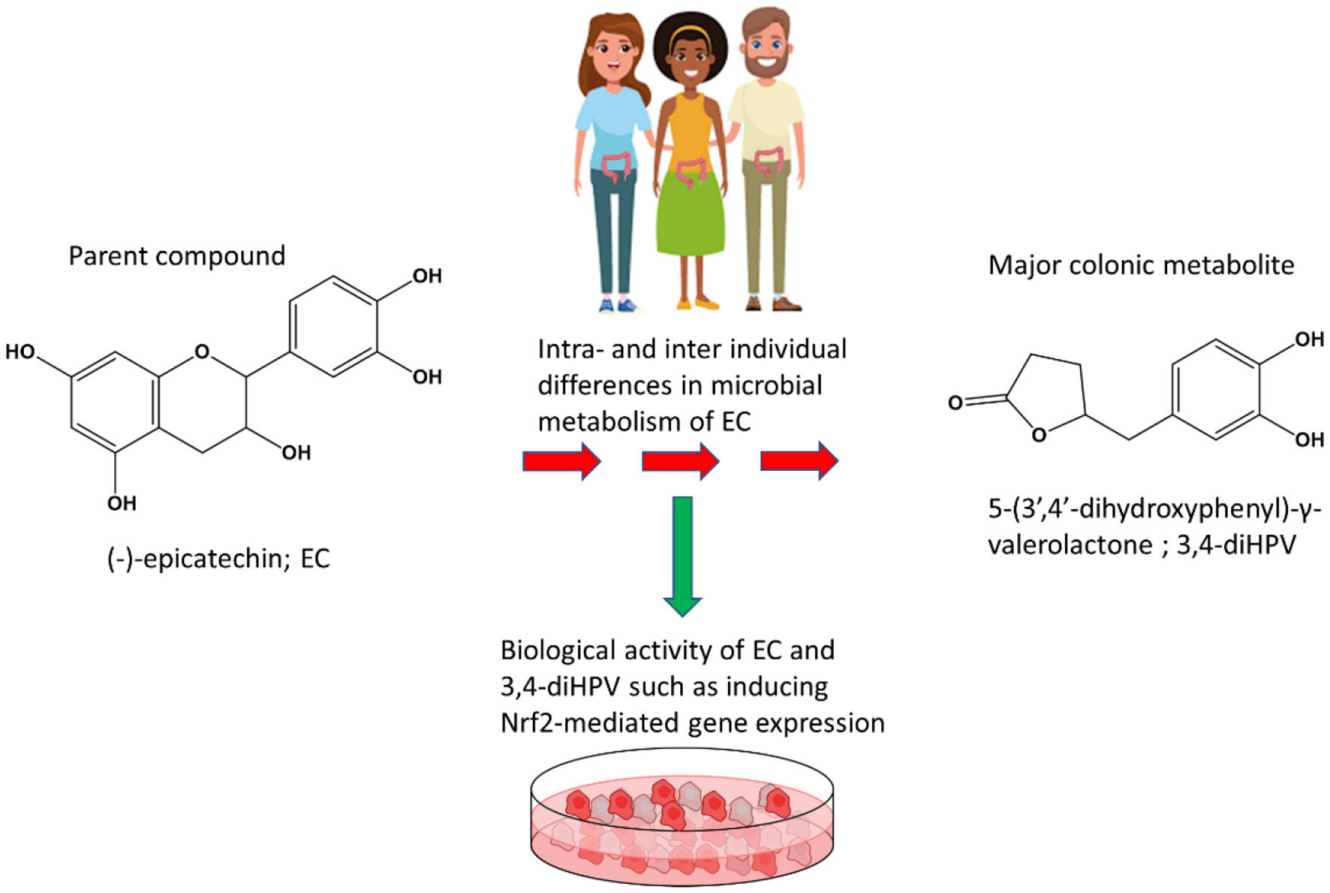


## Introduction

The (-)-Epicatechin (EC) is one of the most common dietary catechins known to occur widely in various food sources, e.g., cocoa, green tea, wine, apple, etc. (1, 2). EC consumption has been linked to a series of beneficial health effects such as antioxidant, anti-inflammatory, anti-cancer, and anti-cardiovascular disease activities (3–6). These pharmacological effects of EC have been ascribed to the direct anti-oxidative property of its phenolic hydroxyl groups but, more recently, also to its “indirect” cytoprotective properties *via* activation of the cascade signaling, Kelch-like, and ECH-associated protein 1-NF-E2-related factor 2 (Keap1-Nrf2) signaling pathway, which is crucial in maintaining cellular redox homeostasis (7). Keap1-Nrf2 is considered to represent an important human health hub given its vital regulatory role in many physiological activities (2, 8). Certain polyphenols can react with the thiol groups of Keap1, resulting in the dissociation of Nrf2, thereby preventing Nrf2 from rapid degradation through the proteasome system, and facilitating the nuclear translocation of nascent Nrf2. Ultimately, a diverse array of Nrf2 regulated cytoprotective genes are transcribed, including, for example, UDP-glucuronosyltransferases (UGTs), NAD(P)H quinone dehydrogenase 1 (NQO1), and many others (8, 9).

**Figure 1:**
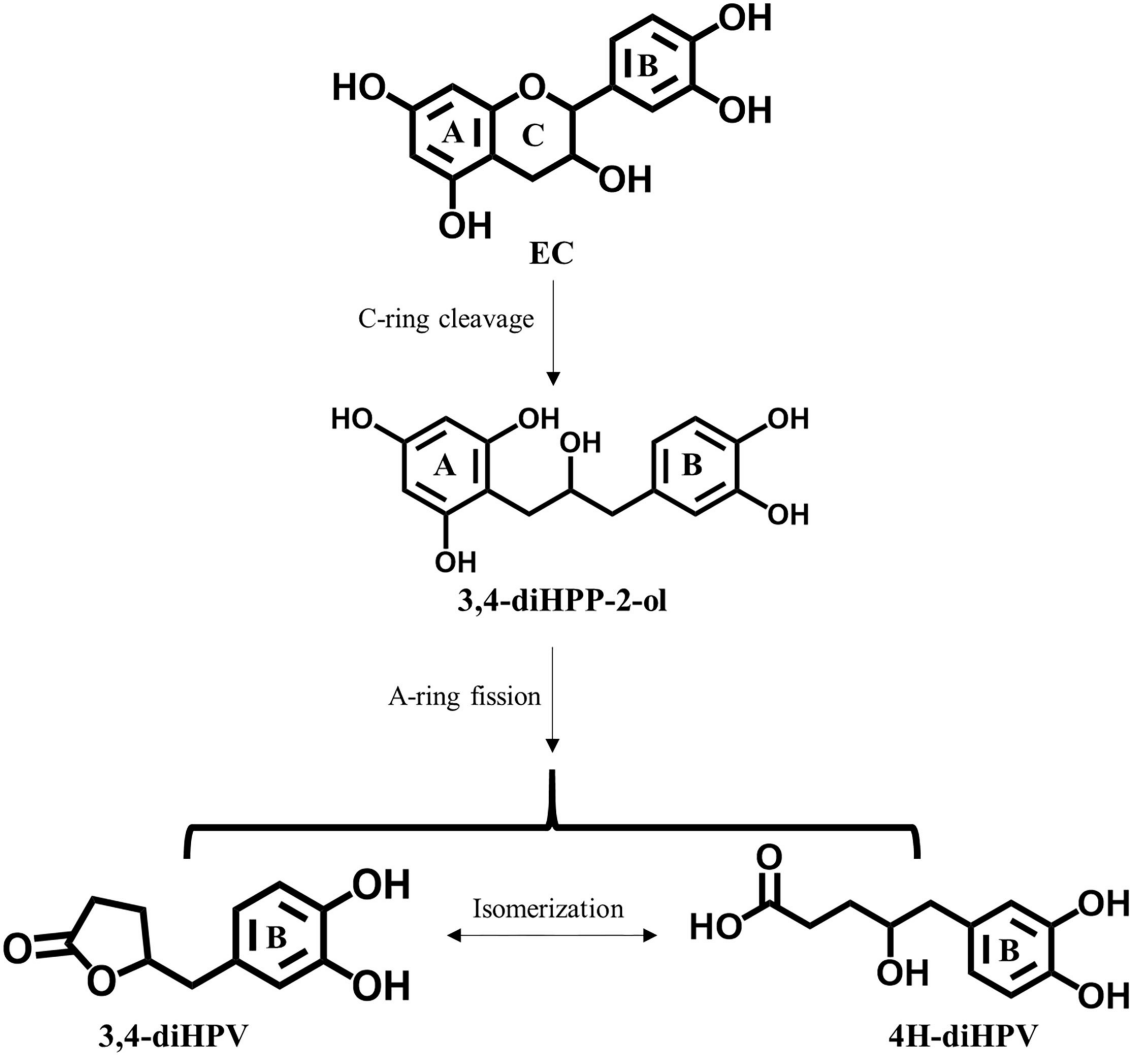
Pathways of human colonic microbial metabolism of Epicatechin (EC).

Even though the daily intake of polyphenols is 10 times that of vitamin C (2), the overall bioavailability of polyphenols is considered to be lower than 10%, resulting in plasma concentration of catechins being more than 50 times lower than those of vitamin C (10–12). EC is recognized as well-absorbed catechin, but still, only about 20% of the intake is absorbed directly in the small gastrointestinal tract and subsequently passed into systemic circulations (12). Over 70% of total EC intake reaches the large intestine where it is subject to extensive degradation by intestinal microbiota (12, 13). Among all the colonic metabolites, 5-(3′,4′-dihydroxy-phenyl-γ-valerolactone (3,4-diHPV) and its downstream metabolites have been reported to account for 42–60% of total EC intake (12, 14). This 3,4-diHPV metabolite results from the A-ring cleavage of the EC C-ring fission intermediate, 1-(3′,4′-dihydroxy-phenyl-3-(2″,4″,6″-trihydroxyphenyl)-2-propanol (3,4-diHPP-2-ol) (Figure 1) (15). Compared with this transient precursor, 3,4-diHPV appeared to accumulate to a substantial level in both *in vitro* and *in vivo* investigations. For instance, valerolactones were reported to persist for a long time in the human or rat body following tea catechin intake. It has also been demonstrated that valerolactones were maximally detected in plasma or urine at 6–24 h (or even longer) following oral intake (or intravenous administration) of different catechins (12, 16–19). The C_max_ of structurally-related EC metabolites in human plasma appeared to occur much earlier, namely, shortly after 1 h following intervention (12). During *in vitro* anaerobic fecal incubations of EC, the metabolite 3,4-diHPV also appeared to be the dominant metabolite formed, with maximum levels observed from 3 to 6 h amounting to 45–49% of the originally incubated concentration of EC, while EC was rapidly degraded already within the first 2 h (20). However, studies on bioactivities of 3,4-diHPV, especially its ability in activating the Keap1-Nrf2 pathway, are still absent.

As is well-known, colonic microbiota play a critical role in the degradation of EC and the formation of 3,4-diHPV. It has been widely accepted that due to the different age, gender, ethnic factors, and diverse lifestyle, the inter-individual microbial compositions could vary substantially (21), hence, affecting the metabolic profiles (20, 22). On the other hand, the microbiota composition is considered to be relatively stable within healthy individuals (23). However, to what extent the inter- and intra-individual differences in microbiota composition can cause differences in the metabolic pattern of conversion of EC still needs to be unveiled.

The aim of the present study was to compare the inter- and intra-individual differences in the metabolic pattern of EC microbial conversion, focusing on the depletion of EC and the formation of 3,4-diHPV, and to better quantify the potential role of this major EC microbial metabolite in inducing Nrf2-mediated gene expression. This will provide novel insights into the health-promoting mechanisms related to dietary EC consumption.

## Materials and Methods

### Chemicals and Reagents

The EC and l-ascorbic acid were ordered from Sigma-Aldrich (Zwijndrecht, the Netherlands). 3,4-diHPV was purchased from BOC Sciences (Shirley, USA). Minimum Essential Medium (MEM), MEM-Alpha, Dulbecco's Modified Eagle Medium with 1:1 F-12 Nutrient Mixture (DMEM/F-12), Penicillin Streptomycin (PS), Penicillin Streptomycin Glutamine (PSG), pyruvate, dextran-coated charcoal-treated fetal calf serum (DCC-FCS), and phosphate-buffered saline (PBS) were obtained from Gibco (Paisley, UK). Fetal calf serum (FCS) was ordered from Bodinco (Alkmaar, the Netherlands). Geneticin (G418), trypsin, and Non-essential amino acids (NEAA) were supplied by Invitrogen Corporation (Breda, the Netherlands).

### Cell Lines

The U2OS Cytotox CALUX cells and the U2OS Nrf2 CALUX cells were both obtained from Biodetection Systems (Amsterdam, the Netherlands). Both cell lines are derived from human osteoblastic cells, transfected with a reporter construct carrying a luciferase reporter gene under transcriptional control of a constitutive promoter or four EpRE sequences (24). Thus, the luciferase expression of U2OS Cytotox cells is invariant and can be used to check the cytotoxicity and luciferase stabilization after exposure to chemicals, enabling the identification of false negatives and false positives, respectively. Meanwhile, the U2OS Nrf2 CALUX cells can be used to measure the luciferase induction after exposure to a potential Nrf2-activator. The cells of both cell lines were cultured in DMEM/F-12 culture medium, supplemented with 10% (v/v) FCS, 1% (v/v) NEAA, and 1% (v/v) PS. The Hepa1c1c7 cells are murine hepatoma cells and were cultured in MEM-Alpha supplemented with 10% (v/v) FCS and 1% (v/v) P/S. To maintain the selection pressure for all three aforementioned cell lines, 200 μg/ml G418 was added to the culture medium during every other subculturing. The Caco-2 cells were obtained from ATCC (Gaithersburg, Maryland, USA) and are derived from a human colorectal adenocarcinoma. They were cultured in MEM culturing medium, supplemented with 20% (v/v) FCS, 1% (v/v) pyruvate, and 1% (v/v) PSG. All cell lines mentioned were cultured in an incubator, at 37°C in a humidified atmosphere with 5% CO_2_.

### Fecal Anaerobic Incubation of EC

Fecal samples were donated by six healthy volunteers, and three different samples were collected at 2-week intervals. These volunteers consisted of two men and four women aged from 25 to 60 years old. No antibiotics have been taken by the volunteers at least 3 months before sampling, and the physical conditions of all volunteers were healthy during the whole sampling period. The collecting and processing of fecal samples and subsequent fecal anaerobic incubations were carried out according to the methodology described previously (20). This methodology was approved by the Medical Ethical Reviewing Committee of Wageningen University (METC-WU) not to require further evaluations based on the Dutch Medical Research Involving Human Subjects Act. In brief, the experimental fecal incubations consisted of 79% (v/v) anaerobic PBS, 20% of the fecal slurries (from different individuals at different visits, final fecal concentration: 40 mg/ml), and 1% (v/v) 10 mM EC (in methanol, final concentration being 100 μM). For the negative control, instead of adding EC, 1% (v/v) methanol was added. The blank control comprised 99% (v/v) PBS and 1% (v/v) 10 mM EC. After 0, 2, 4, 6, and 24 h of anaerobic incubation in the atmosphere of 85% N_2_, 10% CO_2_, and 5% H_2_ at 37°C in a BACTRON300 anaerobic chamber, reactions were terminated by adding one volume of ice-cold methanol followed by putting the sample on ice for 15 min and centrifugation at 21,500 × *g* (VWR, Hitachi Koki Co., Ltd.). In the end, supernatants were collected and stored immediately at −80°C until analysis *via* liquid chromatography triple quadrupole mass spectrometry (LC-TQ-MS). At least three independent incubations were performed for all the different fecal samples.

### LC-TQ-MS Analysis

The EC and 3,4-diHPV contents were measured using a Shimadzu LC-TQ-MS 8045 (Shimadzu, Benelux, B.V. the Netherlands). A previously optimized method was used to qualitatively and quantitatively identify the two compounds (20). Briefly, mobile phase A was composed of water: acetic acid (999: 1, v/v), and mobile phase B was methanol. The flow rate was 0.4 ml/min with the following gradient: 0–0.5 min: 5% B, 0.5–5 min: 5–25% B, 5–6 min: 25–100% B, 6–7 min: 100% B, 7–8 min: 100–5% B, 8–13 min: 5% B. Authentic chemical standards of EC and 3,4-diHPV were used for identification and defining calibration curves for quantifications. Since there was no degradation of EC detected in blank control samples (data not shown), concentrations of compounds in the incubated samples were calculated by subtracting the concentrations quantified in corresponding negative controls incubated without adding EC.

### Reporter Gene Assay

The activity of EC and 3,4-diHPV in inducing Nrf2 gene transcription was determined using the U2OS Nrf2 CALUX reporter gene assay. The method was performed essentially as previously described (22). Briefly, U2OS Nrf2 CALUX cells were cultured in a black 96-well microplate with a clear bottom (Greiner Bio-One) at a density of 2 × 10^5^ cells/ml (2 × 10^4^ cells in 100 μL culture medium per well) for 24 h to let the cells attach to the bottom. The culture medium was then replaced with an exposure medium containing model compounds at different concentrations to expose the cells for another 24 h. In total, eight exposure concentrations were applied for both EC and 3,4-diHPV, ranging from 15 to 150 μM, added from 200 times concentrated stock solutions in Dimethyl Sulfoxide (DMSO). After exposure, the luminometer (GloMax-Multi Detection System-Promega) was used to measure the luciferase activity of each well. The relative light units (RLUs) of all the wells were measured after the automatic injection of 100 μl flash mix (2.67 mM MgSO_4_, 1.07 mM (MgCO_3_)_4_ Mg (OH)_2_ · 5H_2_O, 20 mM Tricine,0.1 mM EDTA, 5 mM ATP; 2 mM DTT, 470 μM D-luciferin, pH 7.8).

In addition, a paralleled U2OS Cytotox CALUX assay was performed to investigate the potential cytotoxicity and whether luciferase stabilization occurred during the exposure to EC or 3,4-diHPV. This assay was performed using the same conditions as described above for the U2OS Nrf2 CALUX assay. Results are shown as the induction factor (IF) compared to the solvent control. At least three independent assays were carried out for both test compounds.

### Sample Preparation and Protein Identification

A label-free relative protein quantitation was performed to characterize the protein level change in EC or 3,4-diHPV treated cells. The identification and relative quantification were conducted *via* a Thermo EASY nanoLC 1000 (Thermo, Waltham, MA, USA) coupled with an Orbitrap Exploris 480 (Thermo electron, San Jose, CA, USA). Both Hepa1c1c7 cells and Caco-2 cells were seeded in T25 flasks at a density of 6.25 × 10^4^ cells/cm^2^ for 24 h. Subsequently, the culture medium was replaced with an exposure medium containing 30 μM EC or 3,4-diHPV in the presence of 0.5 mM l-ascorbic acid for a continuous 24 h exposure. The inclusion of l-ascorbic acid in the exposure medium is not only because it is highly abundant in human plasma but also is reported to be able to improve the stability of polyphenols (11, 25, 26). The selection of the test compound concentration considered the results of both a WST-1 assay (Supplementary Figure S1 in Support Information 1) and the U2OS Cytotox CALUX reporter gene assay, to make sure that non-cytotoxic concentrations were tested. After exposure, the cells were washed two times with PBS, and 1.5 ml of 100 mM Tris-HCl, with pH 8 was added. Afterward, the cells were scraped off and transferred to 2-ml Eppendorf low binding tubes for centrifuging at 1 × 10^4^ rpm (9391 × g) for 3 min. Finally, the cell pellets were dissolved in 100 μL of 100 mM Tris-HCl, with pH 8 and sonicated. Protein concentrations were determined by the Bicinchoninic Acid (BCA) method (27). The protein aggregation capture (PAC) method was applied to obtain peptide samples from protein samples for Thermo EASY nanoLC analysis (28). Peptides of all samples were stored immediately at −20°C until further analysis. Four independent biological replicates were collected for all treatments.

The LC-MS/MS parameters were adapted from a previous chromatographic method (29). In brief, 4 μl (Caco-2 cell samples) or 5 μl (Hepa1c1c7 cell samples) peptide samples were loaded onto an in-house prepared 0.10 × 250 mm ReproSil-Pur 120 C18-AQ with 1.9-μm beads analytical column at a constant pressure of 825 bar. Mobile phase A was 0.1% formic acid in water (v/v), and mobile phase B was 0.1% formic acid in acetonitrile (v/v). The flow rate was 0.5 μl/min with a 50 min linear gradient from 9 to 34% mobile phase B. An electrospray potential of 3.5 kV was applied directly to the eluent *via* a stainless-steel needle fitted into the waste line of a micro cross that was connected between the nLC and the analytical column. MS and MSMS AGC targets were set to 300 and 100%, respectively, or maximum ion injection times of 50 ms (MS) and 30 ms (MSMS) were used. Higher energy collisional dissociation (HCD) fragmented (isolation width 1.2 m/z, 28% normalized collision energy) MSMS scans in a cycle time of 1.1 s/of the most abundant 2–5+ charged peaks in the MS scan were recorded in data-dependent mode (resolution 15,000, threshold 2e4, with 15 s exclusion duration for the selected m/z ± 10 ppm).

The MaxQuant software package (1.6.3.4) was used to identify and, relatively, quantify the peptides (30). Human (UP000005640) and Mouse (UP000000589) databases were downloaded from Uniprot, which was used together with a contaminants database that contains sequences of common contaminants like Trypsins (P00760, bovine and P00761, porcin) and human keratins [Keratin K22E (P35908), Keratin K1C9 (P35527), Keratin K2C1 (P04264), and Keratin K1CI (P35527)]. The “label-free quantification,” as well as the “match between runs” options, were enabled. Deamidated peptides were allowed to be used for protein quantification and all other quantification settings were kept default. Data were filtered to show only proteins reliably identified by minimally 2 peptides, of which at least one is unique, and one is unmodified using a false discovery rate (FDR) of <1% on both protein and peptide levels. After grouping sample replicate injections, an extra filtering step, leaving only protein groups consisting of minimally two label-free quantitation (LFQ) intensities in at least one group, was performed. A normal log was applied to all LFQ intensities. All the remaining non-existing LFQ intensities were replaced by a value that is slightly lower than the lowest measured value. For the Hepa1c1c7 cells, this was done by replacing the values from a normal distribution with default settings (width = 0.3, down shift = 1.8), and for the Caco-2 cells samples, a constant value of 6.6 was used.

### Data Analysis and Bioinformatics

From the derived protein expression profiles, Nrf2-associated proteins were selected manually based on literature information (8, 9, 31–33). The protein expression fold changes (FCs) were calculated as the ratio of normalized label-free quantitation (LFQ) intensity of a specific treatment and that of the solvent control. Besides this dedicated protein dataset analysis, a general proteomics bioinformatical analysis was also performed. The statistical program Perseus (Max Planck Institute, Germany) was used for filtering and a Student's *t*-test for statistical analysis of the changes in the derived protein expression profiles. The differentially expressed proteins (DEPs) were characterized based on meeting two criteria, which are FC < 0.8 or > 1.2 and the *P* < 0.05. Subsequently, the DEPs in EC or 3,4-diHPV treatment were used as input for Gene Ontology (GO) functional classifications and Kyoto Encyclopedia of Genes and Genomes (KEGG) pathway annotation by the DAVID Bioinformatics Resources 6.8 (https://david.ncifcrf.gov/tools.jsp). Furthermore, DEPs were used for protein-protein interaction (PPI) network analysis, which was explored in the STRING database (https://string-db.org/), and the results were further visualized with Cytoscape 3.8.2 (Seattle, USA). Hub protein networks were produced by the Cytoscape 3.8.2 plugin program MCODE.

### Total RNA Isolation and Quantification of MRNA by RT-qPCR

To study the Nrf2-mediated gene expression after exposure to EC or 3,4-diHPV, an RT-qPCR was performed. To this end, both Hepa1c1c7 cells and Caco-2 cells were seeded in a 6-well plate at a density of 6.25 × 10^4^ cells/cm^2^ for 24 h, followed by a continuous 24 h exposure to EC or 3,4-diHPV at 30 μM in the presence of 0.5 mM l-ascorbic acid. Subsequent RNA extraction and purification were carried out with the help of QIAshredder and RNeasy kits (Qiagen, Venlo, the Netherlands). The Nanodrop One (Thermoscientific, Delaware, USA) was used to measure the A260/230, A260/A280, and RNA concentrations. RNA reverse-transcription was performed using the QuantiTect Reverse Transcription Kit (Qiagen, Venlo, the Netherlands) to obtain complementary DNA (cDNA). Gapdh and Actin-beta were chosen as the reference genes for Hepa1c1c7 samples and Caco-2 samples, respectively. The relative transcription levels of Nqo1, Gclc, and Ugt1a6 were determined in Hepa1c1c7 cells and the relative transcription levels of TP53I3, MGST3, and HMOX1 were checked in Caco-2 cells using SYBR Green-based (Qiagen, Venlo, the Netherlands) RT-qPCR. These genes were chosen because they were highly induced in the respective proteomics datasets and/or because they are typical Nrf2-mediated genes. Ct (cycle threshold) values were recorded using a Rotor-Gene 6000 cycler (Qiagen, Venlo, the Netherlands), and used to calculate relative RNA levels by the 2^ΔΔCt^ method. Primers were from the QuantiTect Primer Assays (Qiagen, Venlo, the Netherlands), namely, Mm_Gapdh_3_SG, Mm_Nqo1_1_SG, Mm_Gclc_1_SG, Mm_Ugt1a6_1_SG, Hs_ACTB_1_SG, Hs_TP53I3_va.1_SG, Hs_MGST3_1_SG, and Hs_HMOX1_1_SG.

### Statistical Analysis

Data are presented as mean ± SEM, unless stated otherwise. All experiments were conducted in at least three independent biological replications. The ANOVA or the Student's *t*-test was applied to analyze the statistical significance between treatments and the control group. Statistical significance was determined at *P* < 0.05.

## Results

### Inter- and Intra-Individual Differences in EC Depletion and 3,4-diHPV Formation

**Figure 2:**
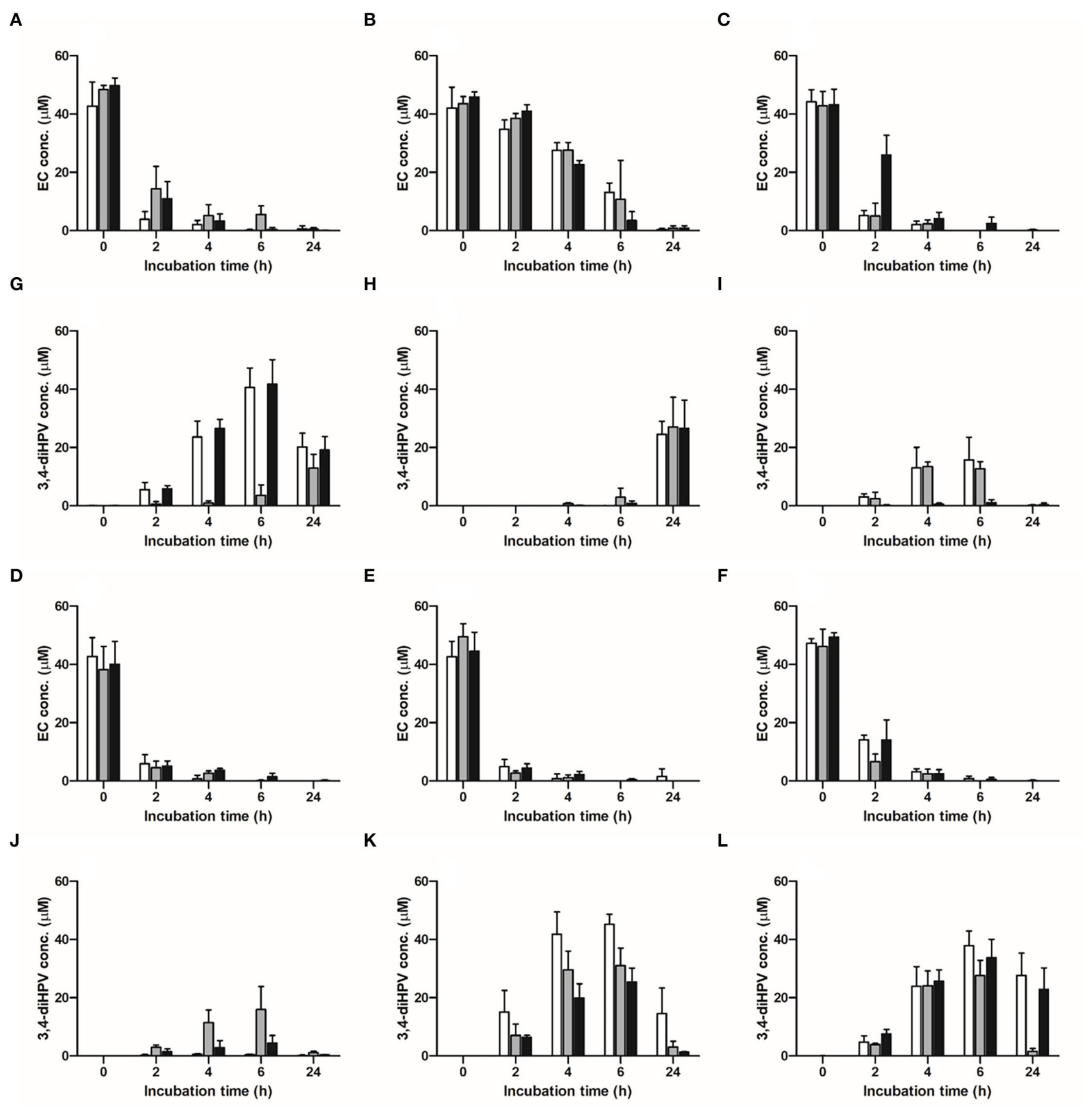
Time-dependent microbial depletion of EC and formation of 3,4-diHPV in anaerobic incubations with fecal samples from six individuals obtained in three collecting weeks. **(A–F)** EC depletion in individuals 1–6; **(G–L)** 3,4-diHPV formation in individuals 1–6. White column: collection 1; gray column: collection 2; black column: collection 3.

The time-dependent microbial depletion of EC and the formation of 3,4-diHPV in anaerobic incubations with fecal samples from six individuals from three independent visits are presented in Figure 2. EC was swiftly degraded after 2 h of incubation except for incubations with the fecal samples from individual 2 (Figure 2B), in which, still 86.9 ± 4.1% of the initial concentration was present. For the incubations with the fecal samples of the other five individuals (Figures 2A,C–F), the average residual EC concentration at 2 h was 18.8 ± 3.5% of the initial concentration, while the initial 50 μM was almost completely degraded at 4 or 6 h. Figures 2G–L show the time-dependent formation of 3,4-diHPV in these fecal incubations. For most incubations, the 3,4-diHPV formation reached a plateau at 4–6 h and decreased after that to show lower levels at 24 h. Also, for 3,4-diHPV the results for individual 2 deviated from the others showing a peak of 3,4-diHPV at 24 h (Figure 2H), in line with this volunteer's relatively slow metabolism of EC (Figure 2B). Figures 2I,J present the time-dependent formation of 3,4-diHPV by fecal samples from individuals 3 and 4, respectively, which were relatively low, amounting to 15.8 and 15.9 μM, respectively, at 6 h, as compared to values that amounted to 27.1–45.2 μM for the samples of the other individuals. This reflects an inter-individual variation in formation and/or further conversion of 3,4-diHPV. This latter explanation was confirmed by the observation that, for example, the metabolite 5-(3′-hydroxyphenyl)-γ-valerolactone (3-HPV) was present in higher amounts in individuals 3 and 4, reaching a peak of 23 μM for individual 3 at 6 h of the collection week 2 compared with only <5 μM for individual 5 in the same condition (data not shown). Besides the differences found between individuals, intra-individual differences were also observed when comparing the results within the individuals for the three fecal samples obtained at different points in time. For instance, only 3.6 μM (7.4% of total mass recovery) 3,4-diHPV was detected in the anaerobic fecal incubation for individual 1 at 6 h for the second sample, while for the third sample, obtained 2 weeks later, this value amounted to 41.8 μM (83.9% of total mass recovery) (Figure 2G).

**Figure 3:**
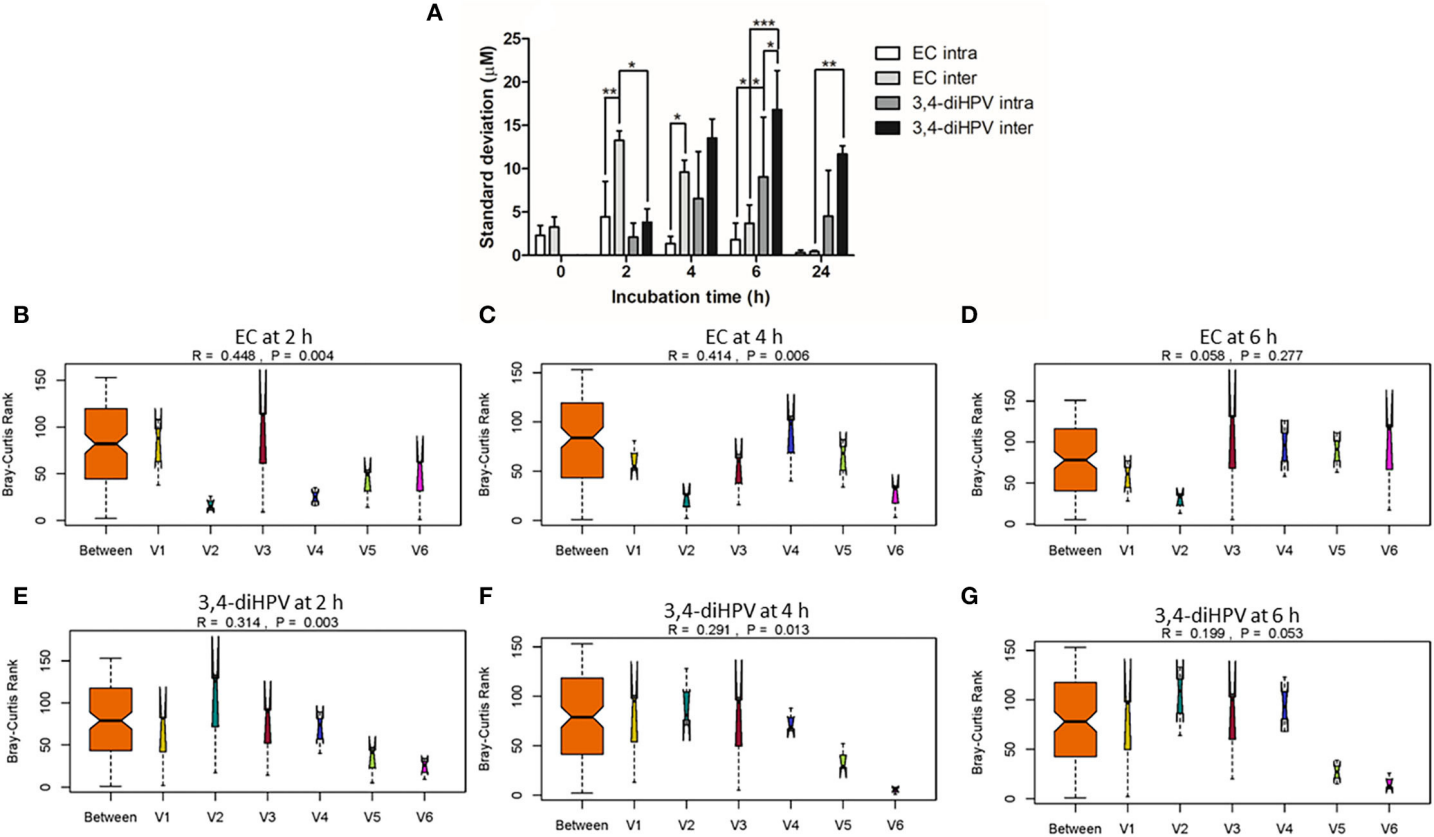
Standard deviations (SDs) that represent intra- and inter-individual differences in EC depletion and 3,4-diHPV formation among six individuals over three collecting weeks **(A)**. Two-way ANOVA with Bonferroni multiple comparisons were performed to evaluate intra- and inter-individual differences in EC depletion and 3,4-diHPV formation. Statistical differences between different treatments are demonstrated (*P < 0.05; **0.001 < *P* < 0.01; ***P < 0.001) **(A)**. Analysis of similarities (ANOSIM) test on results of EC depletion and 3,4-diHPV formation at 2, 4, and 6 h of incubations **(B–G)**; **(B–D)** and **(E–G)** are results of EC depletion and 3,4-diHPV formation at 2, 4, and 6 h, respectively). R > 0 indicates differences between groups are larger than within the group. V1–6: individuals 1–6.

To better characterize the inter- and intra-individual differences in the microbial depletion of EC and formation of 3,4-diHPV, the SDs for the amounts of EC and 3,4-diHPV quantified were calculated within individuals (representing intra-individual differences) and between individuals (representing inter-individual differences), and the results, thus, obtained are presented in Figure 3A. Based on the SDs, the inter-individual differences appeared to be larger than the intra-individual differences at all incubation time points for both the EC and the 3,4-diHPV levels. The highest SD of intra-individual variations for EC and 3,4-diHPV were 4.4 μM (8.8 % of the initial mass) (t = 2 h) and 9.1 μM (18.2 %) (t = 6 h). While the highest SD of inter-individual variations for EC and 3,4-diHPV were 13.3 μM (26.6%) (t = 2 h) and 16.8 μM (33.6%) (t = 6 h) (Figure 3A). A further analysis of similarities (ANOSIM) test was performed to compare within- and between-group similarities of EC depletion and 3,4-diHPV formation at 2, 4, and 6 h of incubations (Figures 3B–G). In line with Figure 3A, the differences between groups (inter-individual differences) are larger than differences within groups (intra-individual differences) for all the analyzed conditions, indicated by R > 0.

### Activation of Nrf2-Mediated Luciferase Expression by EC and 3,4-diHPV

**Figure 4:**
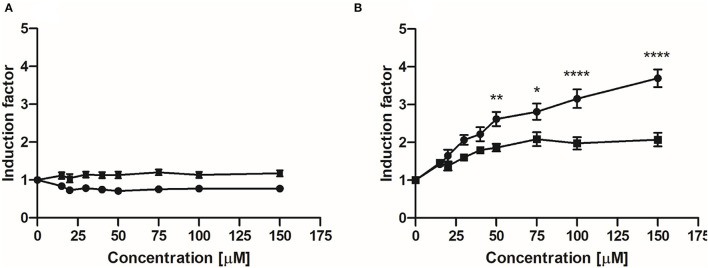
Induction of luciferase expression in U2OS Nrf2 CALUX reporter cells after 24 h exposure to EC **(A)** and 3,4-diHPV **(B)**. Line curves with square symbols represent co-exposure with 0.5 mM l-ascorbic acid (final concentration). While line curves with circular symbols represent exposure with only selected model compounds. Results are presented as mean ± SEM compared with the solvent control, derived from at least three independent experiments. Two-way ANOVA with Bonferroni multiple comparisons were performed to evaluate Nrf2 induction factors induced by 3,4-diHPV, at various concentrations, with and without the presence of 0.5 mM l-ascorbic acid. Statistical differences between different treatments are demonstrated (*P < 0.05; **0.001 < *P* < 0.01; ****P < 0.0001).

Figures 4A,B show the concentration-dependent induction of luciferase expression after 24 h exposure of U2OS Nrf2 cells to EC and 3,4-diHPV, respectively. EC barely showed any activation at all exposure concentrations both in the absence or presence of 0.5 mM l-ascorbic acid (Figure 4A). In contrast, 3,4-diHPV induced significant substantial luciferase induction, with a maximum IF of 2.1 or 3.7 at 150 μM with or without the presence of l-ascorbic acid, respectively (Figure 4B). The results of one-way ANOVA indicate the induction of Nrf2 mediated luciferase expression by 3,4-diHPV was concentration-dependent (Supplementary Figure S2 in Supporting Information 1). Besides, the results also show that the presence of 0.5 mM l-ascorbic acid in the exposure medium significantly reduced the luciferase expression triggered by 3,4-diHPV, especially at higher exposure concentrations of 3,4-diHPV (50–150 μM) (Figure 4B). The results of the U2OS Cytotox CALUX assay confirm that the luciferase induction by 3,4-diHPV is not due to the stabilization of the luciferase reporter protein (Supplementary Figure S3). Moreover, no cytotoxicity was found even at the highest concentration (150 μM) tested for both EC and 3,4-diHPV (Supplementary Figure S3).

### Qualitative and Quantitative Nrf2-Associated Protein Identification

Before initiating the proteomics analysis, the viability of Hepa1c1c7 cells and Caco-2 cells after exposure to EC and 3,4-diHPV for 24 h was analyzed *via* the WST-1 assay. The results show that no significant changes in cell viability were observed at test concentrations up to at least 40 μM (Supplementary Figure S1 in Support Information 1). Subsequently, label-free proteomics was qualitatively and quantitatively identified in a total of 3,991 proteins in Hepa1c1c7 cells and 3,929 proteins and Caco-2 cells, respectively (Supplementary Tables S1, S2 in Support Information 2). When selecting from Nrf2-related proteins (Supplementary Tables S3, S4 in Support Information 2) and applying a selection criterion of a fold change (FC) of >1.2 upon EC or 3,4-diHPV treatment compared to control 17 and 14 proteins were derived from the dataset of the Hepa1c1c7 cells and Caco-2 cells, respectively (Tables 1A,B). The 3,4-diHPV induced more Nrf2-related proteins with higher fold changes than EC both in Hepa1c1c7 cells and Caco-2 cells (Tables 1A,B). Among the 17 Nrf2-related proteins induced in Hepa1c1c7 cells, 14 were induced with a higher FC in the 3,4-diHPV treated cells as compared to the EC treated cells, while only three proteins showed a higher FC upon EC treatment than upon 3,4-diHPV exposure of the cells (Table 1A). For instance, both Nqo1 and Nqo2 proteins were upregulated with a 3.6 and 1.3 higher FC upon 3,4-diHPV as compared to the EC exposure, respectively. Similarly, for the Nrf2-related proteins in the Caco-2 dataset, 3,4-diHPV was also more inducive than EC, with 10 proteins showing higher FCs in 3,4-diHPV exposed cells than in the EC group, while again only four proteins showed higher FCs in the EC group than in the 3,4-diHPV group (Table 1B).

**Table 1:** A comparison of fold change (FC) of Nrf2-associated proteins after exposure to EC and 3,4-diHPV for 24 h at 30 μM.

**(A) Hepa1c1c7 cell samples**				
**Protein IDs**	**Protein names**	**Gene names**	**EC**	**3,4-diHPV**
Q64669	NAD(P)H dehydrogenase [quinone] 1	Nqo1	1.15 (0.901)	3.60 (0.138)
Q9JI75	Ribosyldihydronicotinamide dehydrogenase [quinone]	Nqo2	1.08 (0.294)	1.31 (0.041)
Q8CHT0	Aldehyde dehydrogenase family 4 memberA1	Aldh4a1	1.21 (0.317)	1.46 (0.045)
D3Z0B9	Aldehyde dehydrogenase family 16 member A1	Aldh16a1	1.03 (0.976)	1.73 (0.384)
P19157	Glutathione S-transferase P 1	Gstp1	1.26 (0.018)	1.57 (0.001)
P97494	Glutamate–cysteine ligase catalytic	Gclc	1.13 (0.374)	1.36 (0.026)
P09671	Superoxide dismutase (10), mitochondrial	Sod2	1.06 (0.733)	1.28 (0.252)
P10639	Thioredoxin	Txn	1.10 (0.166)	1.36 (0.015)
Q9JMH6	Thioredoxin reductase 1, cytoplasmic	Txnrd1	1.20 (0.419)	1.52 (0.179)
J3QMN4	Thioredoxin reductase 2, mitochondrial	Txnrd2	1.17 (0.350)	1.46 (0.066)
A0A1L1STE6	Isocitrate dehydrogenase (56) subunit, mitochondrial	Idh3a	1.19 (0.388)	1.28 (0.210)
P70404	Isocitrate dehydrogenase (56) subunit gamma 1, mitochondrial	Idh3g	1.24 (0.265)	1.20 (0.330)
P61222	ABCE1	Abce1	1.01 (0.916)	1.26 (0.074)
Q61753	D-3-phosphoglycerate dehydrogenase	Phgdh	1.05 (0.608)	1.20 (0.089)
P39689	Cyclin-dependent kinase inhibitor 1	Cdkn1a	2.32 (0.513)	6.16 (0.093)
P28033	CCAAT/enhancer-binding protein beta	Cebpb	1.20 (0.427)	1.04 (0.868)
O54790	Transcription factor MafG	Mafg	1.23 (0.249)	1.06 (0.718)
**(B) Caco-2 cell samples**				
Q53FA7	Quinone oxidoreductase PIG3	TP53I3	1.74 (0.577)	3.53 (0.130)
Q9Y6N5	Sulfide:quinone oxidoreductase	SQRDL	2.93 (0.237)	2.05 (0.416)
Q04828	Aldo-keto reductase family 1 member C1	AKR1C1	2.10 (0.456)	2.94 (0.291)
Q14914	Prostaglandin reductase 1	PTGR1	2.04 (0.420)	2.43 (0.320)
O75223	Gamma-glutamylcyclotransferase	GGCT	2.21 (0.455)	2.72 (0.344)
Q8TED1	Probable glutathione peroxidase 8	GPX8	2.11 (0.376)	0.97 (0.979)
Q13162	Peroxiredoxin-4	PRDX4	1.57 (0.034)	1.24 (0.410)
P19224	UDP-glucuronosyltransferase	UGT1A6	1.10 (0.749)	1.31 (0.344)
P34913	Bifunctional epoxide hydrolase 2	EPHX2	1.14 (0.440)	1.40 (0.043)
Q13501	Sequestosome-1	SQSTM1	1.05 (0.969)	1.21 (0.874)
O75027	ABCB7	ABCB7	0.49 (0.526)	2.31 (0.297)
Q9NRK6	ABCB10	ABCB10	1.07 (0.763)	1.30 (0.249)
Q9NP58	ABCB6	ABCB6	1.31 (0.027)	1.28 (0.007)
O15439	ABCC4	ABCC4	1.16 (0.643)	1.41 (0.282)

*A selection criterion of FC > 1.2 in either EC or 3,4-diHPV treatment compared with control was applied. (A,B) were derived from Hepa1c1c7 cells and Caco-2 cells, respectively (numbers in parentheses are p-values)*.

### Identification of Statistically Significant Differentially Expressed Proteins (DEPs) and Functional Annotations

**Figure 5:**
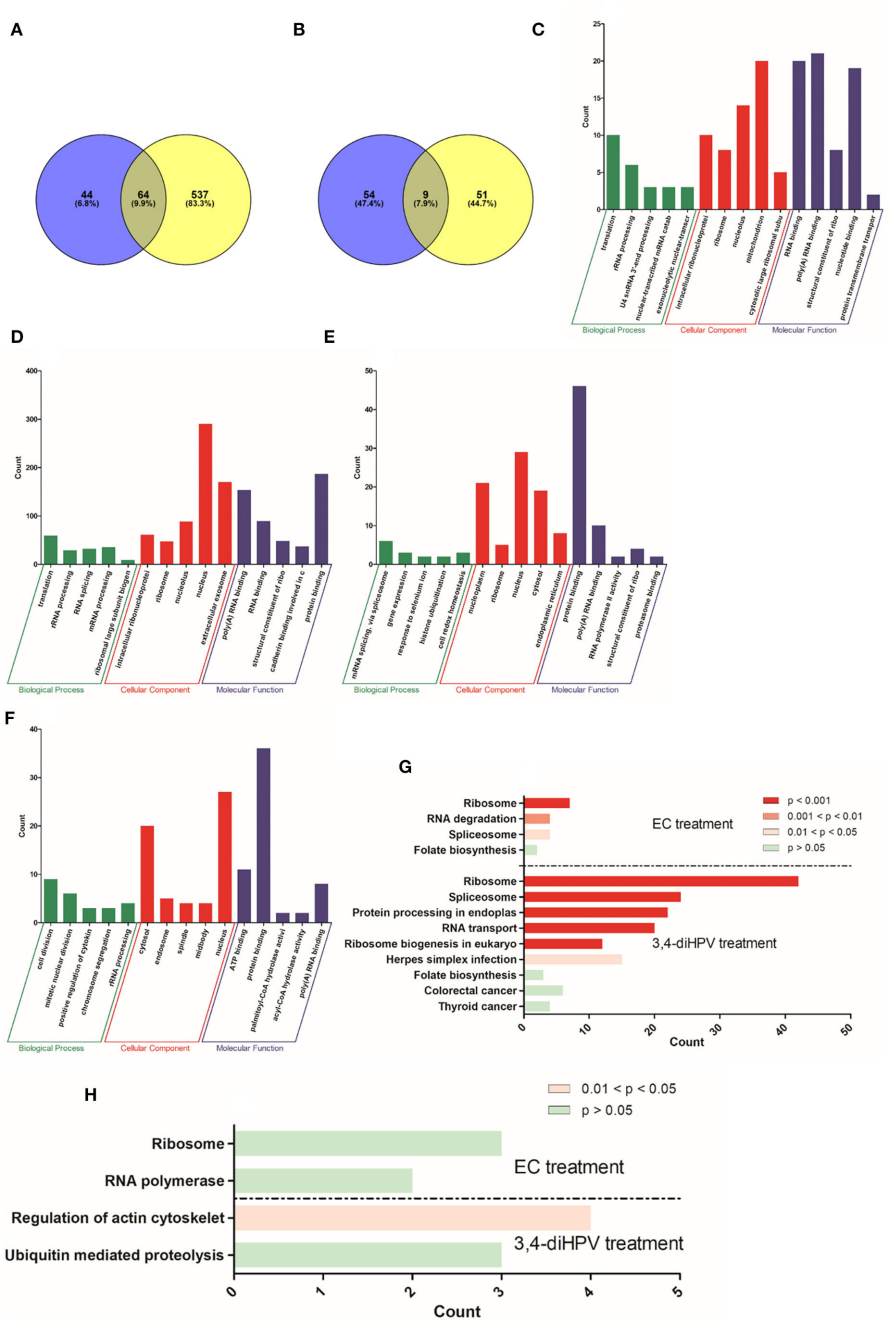
Numbers of DEPs identified by label-free proteomics upon treatment of Hepa1c1c7 cells **(A)** and Caco-2 cells **(B)** with either 30 μM EC (blue circle) or 30 μM 3,4-diHPV (yellow circle). GO enrichment of DEPs from 30 μM EC **(C)** and 30 μM 3,4-diHPV **(D)** treated Hep1c1c7 cells and from 30 μM EC **(E)** and 30 μM 3,4-diHPV **(F)** treated Caco-2 cells. The y-axis value reflects the number of proteins differentially regulated in respective GO terms. KEGG pathway enrichment analysis of DEPs from Hepa1c1c7 cells **(G)** and Caco-2 cells **(H)** treated with 30 μM of either EC or 3,4-diHPV.

To further obtain more insights into changes in protein profiles upon exposure of Hepa1c1c7 cells or Caco-2 cells to either EC or 3,4-diHPV, statistically significant differentially expressed proteins (DEPs) were derived from both EC and 3,4-diHPV treated Caco-2 cells and Hep1c1c7 cells using two criteria which were FC < 0.8 or > 1.2 and the *P* < 0.05 (Figure 5). The protein dataset of Hepa1c1c7 cells revealed 108 and 601 DEPs characterized upon EC and 3,4-diHPV treatments, respectively, with 64 of these DEPs being shared for both treatments (Figure 5A). Meanwhile, the Caco-2 protein dataset, revealed 63 and 60 DEPs, upon exposure to EC and 3,4-diHPV, respectively, with nine DEPs in common between both treatments (Figure 5B).

Subsequently, DEPs were used to perform a GO enrichment to determine their role in the categories such as biological processes (BPs), cellular components (CCs), and molecular functions (MFs). Full GO enrichment analysis results can be found in Support Information 2. Figures 5C–F depict the top five most significantly enriched terms in the three different categories after exposure of Hepa1c1c7 cells and Caco-2 cells to EC and 3,4-diHPV. The x-axis presents different enriched terms and the y-axis indicates the number of proteins that fell into the enriched terms. Several mutual terms were obtained for EC and 3,4-diHPV treated Hep1c1c7 cells (Figures 5C,D). For example, translation and rRNA processing of the category BP were enriched after both treatments. In the category CC, the DEPs were enriched in intracellular ribonucleoprotein complex, ribosome, and nucleolus for both treatments. MF comprised poly(A) RNA binding, RNA binding, and structural constituent of the ribosome in both treatments (Figures 5C,D). This result suggests that EC and 3,4-diHPV stimulated some comparable cellular responses in Hepa1c1c7 cells and translation-related activities were likely to be affected most in both EC and 3,4-diHPV groups. Moreover, the EC exposure resulted in most protein changes in mitochondria, while 3,4-diHPV exposure caused the most protein changes in the nucleus, also suggesting differences in the target sites of the two compounds. Additionally, in line with the fact that more DEPs were characterized in the 3,4-diHPV group, the protein counts in its enriched terms for the BP, CC, and MF categories were also higher than those for the EC treatment (Figures 5C,D). In contrast, in Caco-2 cells, not many terms were enriched upon either EC or 3,4-diHPV exposure. The data showing that only nucleus in the terms of CC and poly(A) RNA binding in the category of MF were mutually enriched after both EC and 3,4-diHPV treatments (Figures 5E,F).

Furthermore, to better understand to which signaling pathways and metabolic activities the DEPs contribute, a KEGG pathway enrichment analysis was conducted for the Hepa1c1c7 and Caco-2 datasets. Figure 5G describes the enriched pathways in Hepa1c1c7 cells. Ribosome and Spliceosome were significantly enriched both upon EC and 3,4-diHPV treatments. Folate biosynthesis was also found to be enriched in the two groups, albeit not to a statistically significant extent (Figure 5G). On the other hand, the pathways enriched in Caco-2 cells were very limited. Only two pathways were obtained for each treatment while regulation of the actin cytoskeleton appeared to represent the only statistically significantly enriched pathway (Figure 5H). From these results, it, thus, also follows that Hepa1c1c7 cells were more responsive to EC and 3,4-diHPV than the Caco-2 cells, and that 3,4-diHPV was more potent in triggering a cellular response. Additionally, to further explore the possible interactions of the DEPs, a protein-protein interaction (PPI) network analysis was conducted, including a hub PPI network analysis, if applicable. Supplementary Figures S4A–D in Support Information 1 show the results of this PPI network analysis also presenting the hub proteins for Hepa1c1c7 DEPs. The derived hub PPI networks (Supplementary Figures S4B,D in Support Information 1) for both treatments indicate that ribosome proteins played important roles among these DEPs, which is in line with the GO enrichment analysis. Supplementary Figures S4E,F in Support Information 1 present the results of a PPI network analysis for Caco-2 DEPs following treatment with EC and 3,4-diHPV, respectively. Fewer PPIs were derived compared to the results obtained for the Hepa1c1c7 cells, while there were no relevant hub proteins found for these two treatments of Caco-2 cells.

### Regulation of Nrf2 Pathway-Related mRNA Expression by EC and 3,4-diHPV

**Figure 6:**
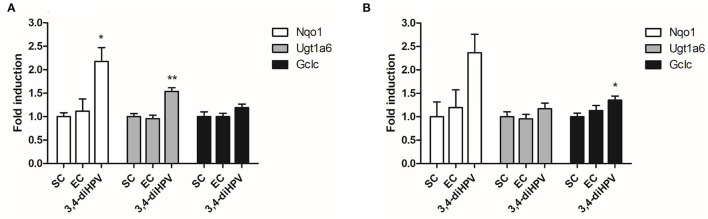
Relative RNA **(A)** and protein **(B)** levels in Hepa1c1c7 cells after exposure to the EC or 3,4-diHPV at 30 μM. Gene/protein names are depicted as figure legends. The results were calculated as the ratio of the treatment and the solvent control (SC) and are presented as mean ± SEM, derived from at least three independent experiments. Statistical differences between different treatments are demonstrated (*P < 0.05; **P < 0.01).

To corroborate the potential Nrf2-activation effects of EC and 3,4-diHPV, RT-qPCR was performed for confirmation of some Nrf2-regulated gene expression changes after exposure. Figure 6A presents the results for the relative RNA level for Nqo1, Ugt1a6, and Gclc in Hepa1c1c7 cells after exposure to 30 μM EC or 3,4-diHPV. EC exposure did not significantly affect the RNA level of any of the three genes in Hepa1c1c7 cells. While 3,4-diHPV significantly upregulated the RNA level of Nqo1 and Ugt1a6 by 2.2- and 1.5-fold, respectively. In 3,4-diHPV, exposed Hepa1c1c7 cells Gclc was slightly increased (1.2-fold), albeit not statistically significant. These results are well in line with the proteomics data. The fold change (FC) of NAD(P)H dehydrogenase [quinone] 1, UDP-glucuronosyltransferase 1, and glutamate cysteine ligase catalytic subunit (the corresponding proteins of the three genes) amounted to 2.4, 1.2, and 1.4 upon 3,4-diHPV treatment Hepa1c1c7 cells (Figure 6B). While their FCs in EC treatment were between 1 and 1.2, indicating no obvious alterations at the protein level (Figure 6B). On the other hand, the relative RNA levels of TP53I3, MGST3, and HMOX-1 in Caco-2 cells were not significantly changed after either EC or 3,4-diHPV exposure, suggesting that in these cells, the Nrf2 pathway may not be activated (Supplementary Figure S5).

## Discussion

In this study, we have compared the inter- and intra-individual differences in human colonic microbial metabolism of EC. Subsequently, the bioactivity for activating Nrf2 signaling of both the parent compound EC and its dominant colonic metabolite 3,4-diHPV were investigated.

Polyphenols are considered important micronutrients and are abundant in our diets. However, their bioavailability is low, as evidenced by the fact that their maximum plasma concentrations rarely exceed 1 μM after consumption of 80–100 mg quercetin equivalent (34, 35). Following the limited bioavailability, substantial amounts of non-absorbable polyphenols are known to reach the colon where they are subject to further gut microbial transformations, including ring fission, dihydroxylation, decarboxylation, etc. The host microbial composition is known to vary substantially among people due to differences in age, gender, ethnic factors, lifestyle, and diet, which, in turn, could cause inter-individual differences in gut microbial metabolism of polyphenols (20, 36–38). Unlike the inter-individual differences, the intra-individual differences in colonic microbial composition and resulting metabolic conversions have scarcely been studied, as it is believed that the intestinal microbiota composition of an individual can be steady for years (39, 40).

The result of the time-dependent EC depletion in this study, reveals only limited differences between or within individuals, with the data for individual 2, who appeared to be a slow metabolizer, showing only about 4-fold lower conversion at 2 h fecal incubation time than what was observed for the other five individuals. This could be a reflection of the functional redundancy of gut microbiota, providing the possibility for a reaction to be catalyzed by multiple microbiota (41). The formation of 3,4-diHPV from EC, which includes several reaction steps and reflects a balance between its formation and subsequent further conversion, showed larger inter- and intra-individual differences, than the decrease of EC, suggesting the reactions require specific groups of microbiotas. So far, *Lactobacillus plantarum, Eggerthella lenta, Adlercreutzia equolifaciens*, and *Eubacterium SDG-2* are the only identified microbiota that can open the C-ring of catechins to form diphenylpropan-2-ol, the precursor of 3,4-diHPV (42–45). Subsequently, diphenylpropan-2-ol is converted into its A-ring fission metabolites, 3,4-diHPV and 4-hydroxy-5-(3′,4′-dihydroxyphenyl)-valeric acid (4H-diHPVA), and this step is reported to be mediated by *Flavonifractor plautii* (44). Differences in the time-dependent formation of 3,4-diHPV were also reported in a recently published study by Li et al. (45). They studied *in vitro* microbial metabolism of (+)-catechin, and the major metabolite 3,4-diHPV was also found to peak at different time points in different individuals, e.g., 24 h for individual 1, 48 h for individual 6, and 72 h for individual 7. By establishing correlations between microbial metabolic rate of catechin and the individual microbiota compositions, they discovered that faster metabolizers had a significantly higher prevalence of, e.g., *Eggerthella* and *Eubacterium*, which are capable of C-ring cleavage of catechin. Therefore, the slower degradation of EC and formation of 3,4-diHPV for individual 2 in this study is probably due the lower abundance of C-ring cleavage and/or A ring fission bacteria in fecal samples of this individual. Interestingly, an obvious different metabolic pattern of 3,4-diHPV formation in individual 1 at collection 2 was found, compared with this individual's collections 1 and 3, with much less 3,4-diHPV detected in the incubations of EC with the fecal sample from the 2nd collection. This indicates the intra-individual gut microbiome can be variable, while the limited differences observed for the fecal samples of the other individuals taken over time reflect the gut microbiome resilience in healthy individuals (21). In the 2nd collection, individual 1's gut microbial ecosystem may have faced perturbations due to environmental factors, such as unhealthy diet, lifestyle, etc. As a result, the metabolic activity appeared different compared with the earlier and later samplings. However, this situation was probably a transient state, not reaching the tipping points, after which the microbial activity recovered to the initial state that also mimicked the final state (39). Collectively, although the inter-individual difference in metabolic pattern appeared larger than the intra-individual differences, it is good to be aware of the possible variations caused by the latter when investigating the role of the gut microbiota in the metabolism of food-borne compounds of interest (46).

Next, we investigated to what extent EC and 3,4-diHPV were able to activate the Nrf2 signaling pathway, often linked to (part of) the beneficial effects of catechin intake (33, 47). The results of the CALUX reporter gene assay suggest that EC, in contrast to its metabolite 3,4-diHPV, was not effective as an Nrf2 activator. This is in agreement with the findings of Muzolf-Panek and colleagues who investigated the induction of EpRE-mediated gene expression by several green tea catechins and found that EC, along with ECG, was inactive at all test concentrations (48). However, Huang and colleagues concluded that EC can protect rats from monocrotaline (MCT) induced liver oxidative injury *via* activating Nrf2 signaling. For instance, they found that administration of 40 mg/kg EC could counteract the effect of MCT exposure of rats on the levels of Gst, Nrf2, Gclc, Gclm, Nqo1, and Hmox-1 (5). This protective effect of EC is likely, based on the findings of the present study, mediated, at least in part, by its microbial metabolite, e.g., 3,4-diHPV, since EC is prone to microbial metabolism in the large intestine in *in vivo* conditions. Moreover, other studies that claimed EC could activate the Nrf2 pathway in cells or rodents were mostly conducted with a pre-stimulated stress model (6, 49, 50), while the present research provides insights into the Nrf2 activation by EC under non-stressed conditions.

Unlike EC, its major colonic metabolite 3,4-diHPV exerted significant induction of Nrf2-mediated luciferase expression in the CALUX reporter gene assay. This may be ascribed to the easier (auto)oxidation of 3,4-diHPV compared to EC to form semiquinone and quinone type metabolites, inducing oxidative and/or electrophilic stress, thus, activating the Nrf2 signaling (8, 51, 52). This was corroborated by the results that the presence of l-ascorbic acid in the exposure medium reduced the induction folds of Nrf2 signaling activation in 3,4-diHPV exposed U2OS Nrf2 CALUX cells since it has been reported that l-ascorbic acid is able to inhibit the auto-oxidation of polyphenols (25, 26, 53). Detection of concentrations of semiquinone- and quinone-type metabolites of EC and 3,4-diHPV in the cell culture medium after exposure remains a topic of interest for further studies.

Following the studies with the U2OS, the Nrf2 CALUX reporter cells' additional studies were performed on the Nrf2-mediated gene and protein expression in cells from the Hepa1c1c7 and the Caco-2 cell lines, providing *in vitro* models for effects in the liver and the colon, respectively. Thus, both the Hepa1c1c7 and the Caco-2 cell lines were used to quantify the EC and 3,4-diHPV-induced RNA and protein level changes. From both RT-qPCR and proteomics results, the Nrf2-mediated genes and proteins were only positively regulated after 3,4-diHPV exposure but not after EC exposure, which is in line with the U2OS Nrf2 CALUX assay result. The DEP responses upon both EC and 3,4-diHPV treatments in Hepa1c1c7 cells were more pronounced than those observed in Caco-2 cells. This may indicate that, compared to colonic cells, hepatic cells are more sensitive to polyphenol-type stimuli. One of the explanations for this apparent difference may be related to the active efflux of polyphenols by the multidrug resistance-associated proteins 2 (MRP2) in Caco-2 cells decreasing the intracellular levels of these compounds (54). Moreover, a substantially higher number (601) of DEPs were obtained in Hepa1c1c7 cells exposed to 3,4-diHPV than when exposed to EC, corroborating the more potent bioactivity of this intestinal microbial metabolite than of the parent catechin. The subsequent bioinformatical analysis also revealed several biological activities of 3,4-diHPV. However, it is worth noting that the Nrf2 pathway or Nrf2-regulated intermediary metabolisms were not among the most significantly enriched pathways indicated by the DEPs, which suggests the Nrf2 signaling may not represent the main target of either EC or 3,4-diHPV. Instead, the translation process was significantly enriched in Hepa1c1c7 cells by both compounds. This is in line with the KEGG enrichment results where spliceosome and ribosome, two important organelles involved in messenger RNA (mRNA) splicing and translation, were highly enriched. These results suggest the potential cell proliferation-promoting effect of EC and 3,4-diHPV at the test concentration. The promotion of cell proliferation by EC was already proven both in *in vivo* and *in vitro* studies and has been linked to the potential cellular protective effects of this compound (55, 56). However, barely any research on 3,4-diHPV regarding this effect is available, so far, thus, providing an interesting topic for future research. Moreover, this metabolite is more bioavailable than its parent compound EC and presents an important biomarker *in vivo* for EC intake (12, 14). It has been reported that valerolactones, together with valeric acids and their phase 2 metabolites, were excreted in quantities equivalent to 42% of the ingested EC, compared with just 20% of EC structurally related metabolites (12, 13).

To conclude, the present study compared the inter- and intra-individual differences in microbial depletion of EC and formation of its major colonic metabolite 3,4-diHPV. Inter-individual differences appeared larger than the intra-individual differences. Unlike its parent compound EC, 3,4-diHPV appeared able to cause a concentration-dependent induction of Nrf2-mediated gene expression in an Nrf2 reporter cell line. Furthermore, hepatic cells seemed to be more responsive than colonic cells to polyphenol exposure. The proteomics data of Hepa1c1c7 cells revealed that 3,4-diHPV was more potent than EC with more DEPs and enriched functional annotations. However, the data also indicated that the Nrf2 pathway may not represent a major target of 3,4-diHPV. Altogether, our results illustrate that the intestinal microbial metabolism of EC produces the important metabolite 3,4-diHPV, which enriches the bioactivities of the parent compound, increasing possibilities for the induction of Nrf2-mediated gene expression. As 3,4-diHPV is a major gut microbiota-derived metabolite of EC, both intra- and inter-differences in its formation from EC could affect the health-beneficial effects of EC consumption.

## Data Availability Statement

The mass spectrometry proteomics data have been deposited to the ProteomeXchange Consortium via the PRIDE partner repository with the dataset identifier PXD033248.

## Author Contributions

CL and SB performed experiments and interpreted data. CL wrote the manuscript. IR designed the scope of the manuscript and revised the manuscript. All authors read and approved the final manuscript.

## Funding

CL was grateful for the financial support of the China Scholarship Council (CSC) (Grant Number: 201803250053).

## Conflict of Interest

The authors declare that the research was conducted in the absence of any commercial or financial relationships that could be construed as a potential conflict of interest.

## Publisher's Note

All claims expressed in this article are solely those of the authors and do not necessarily represent those of their affiliated organizations, or those of the publisher, the editors and the reviewers. Any product that may be evaluated in this article, or claim that may be made by its manufacturer, is not guaranteed or endorsed by the publisher.

## Supplementary Material

Cell viability results from WST-1 assay (Supplementary Figure S1). One-way ANOVA for U2OS Nrf2 CALUX assay (Supplementary Figure S2). U2OS Cytotox CALUX assay (Supplementary Figure S3). PPI network analysis (Supplementary Figure S4). Relative RNA and protein levels in Caco-2 cells after exposure to EC or 3,4-diHPV (Supplementary Figure S5). The proteins identified and quantified in Hepa1c1c7 and Caco-2 cells (Supplementary Tables S1, S2). Nrf2-associated proteins selected from whole protein datasets of Hepa1c1c7 and Caco-2 cells, respectively (Supplementary Tables S3, S4). GO and KEGG pathway enrichment results in Hepa1c1c7 and Caco-2 (Support Information 2).
