# Supplementary material for: Intra- and Inter-individual Differences in the Human Intestinal Microbial Conversion of (-)-Epicatechin and Bioactivity of Its Major Colonic Metabolite 5-(3′,4′-Dihydroxy-Phenyl)-γ-Valerolactone in Regulating Nrf2-Mediated Gene Expression
Source: Front Nutr. 2022 Jun 30;9:910785. doi: 10.3389/fnut.2022.910785 (PMC9281540; doi:10.3389/fnut.2022.910785)
Supplement: Supplementary file 1 [file Data_Sheet_1.docx]

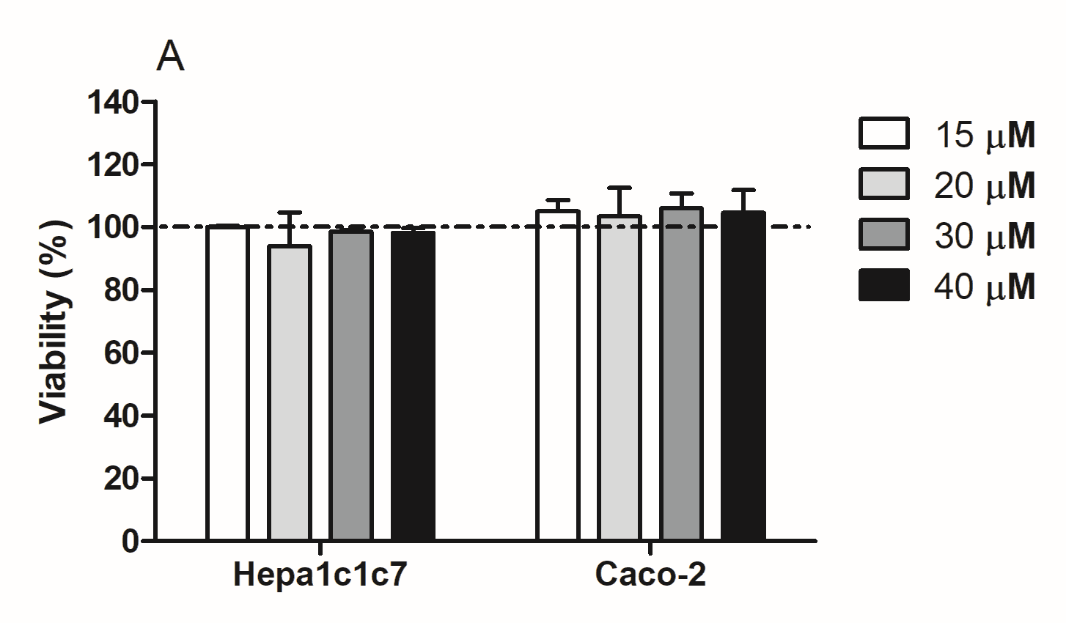


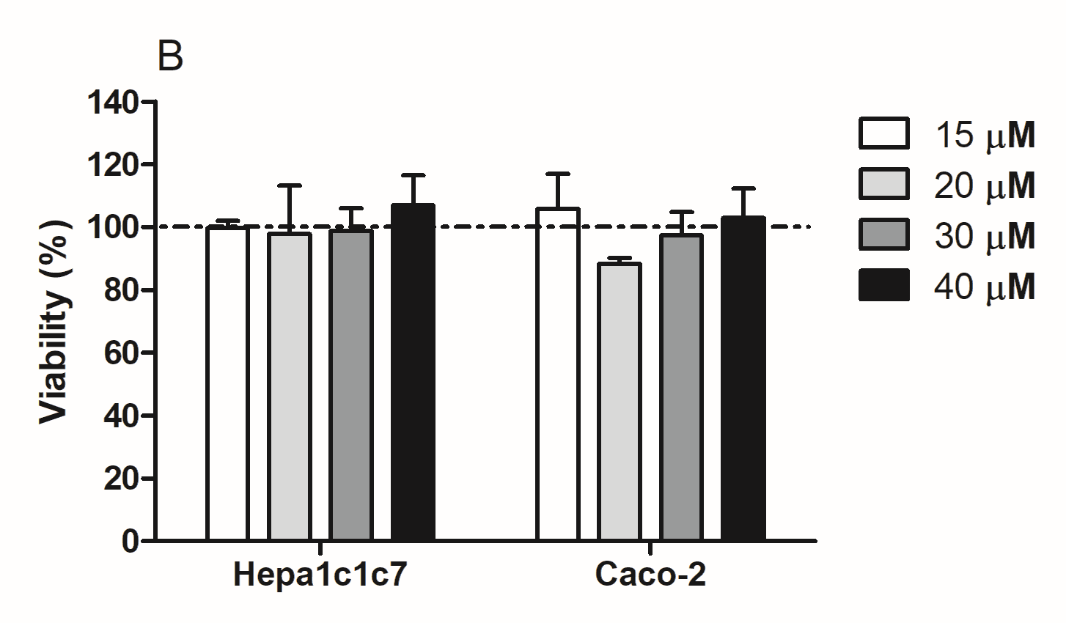


**Figure S1.** Cell viability test of Hepa1c1c7 cells and Caco-2 cells exposed to EC (A) and 3,4-diHPV (B) at different concentrations, conducted via WST-1 assay. All exposure with the presence of 0.5 mM ascorbic acid. The results are presented as mean ± SEM compared with solvent control, derived from at least three independent experiments.


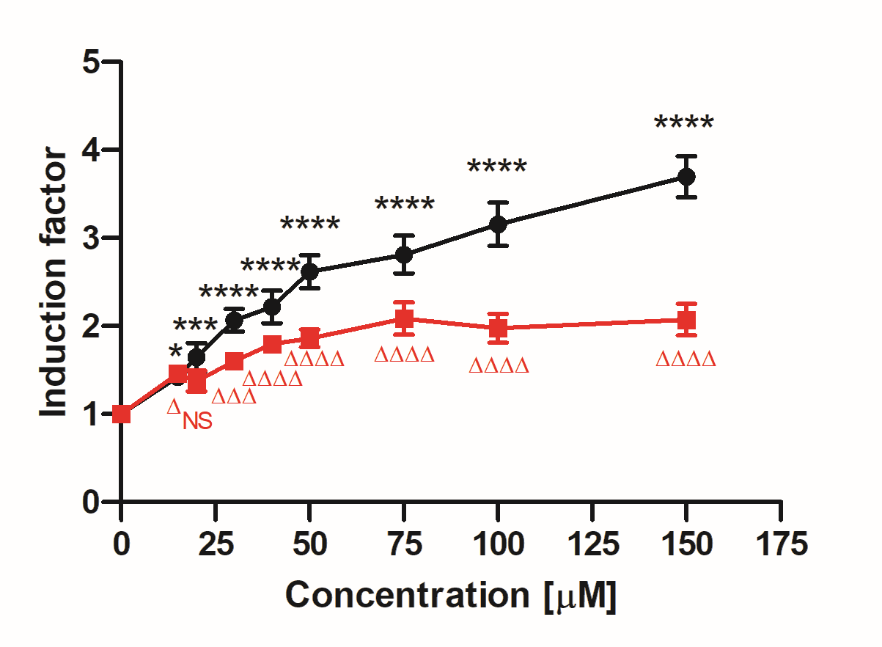


**Figure S2.** Induction of luciferase expression in U2OS Nrf2 CALUX reporter cells after 24 h exposure to 3,4-diHPV. Line curves with red square symbols represent co-exposure with 0.5 mM L-ascorbic acid (final concentration). While line curves with black circular symbols represent exposure with only 3,4-diHPV. Results are presented as mean ± SEM compared with the solvent control, derived from at least three independent experiments. One-way ANOVA with Bonferroni multiple comparisons with the repective solvent control were performed to evaluate the concentration-dependent Nrf2 mediated luciferiase exrepssion induced by 3,4-diHPV in the presence or absence of L-ascorbic acid. Statistical differences between different treatments are demonstrated (* or Δ, P < 0.05; *** or ΔΔΔ, 0.01 < P < 0.001; **** or ΔΔΔΔ, P < 0.0001; NS, not significant).

**
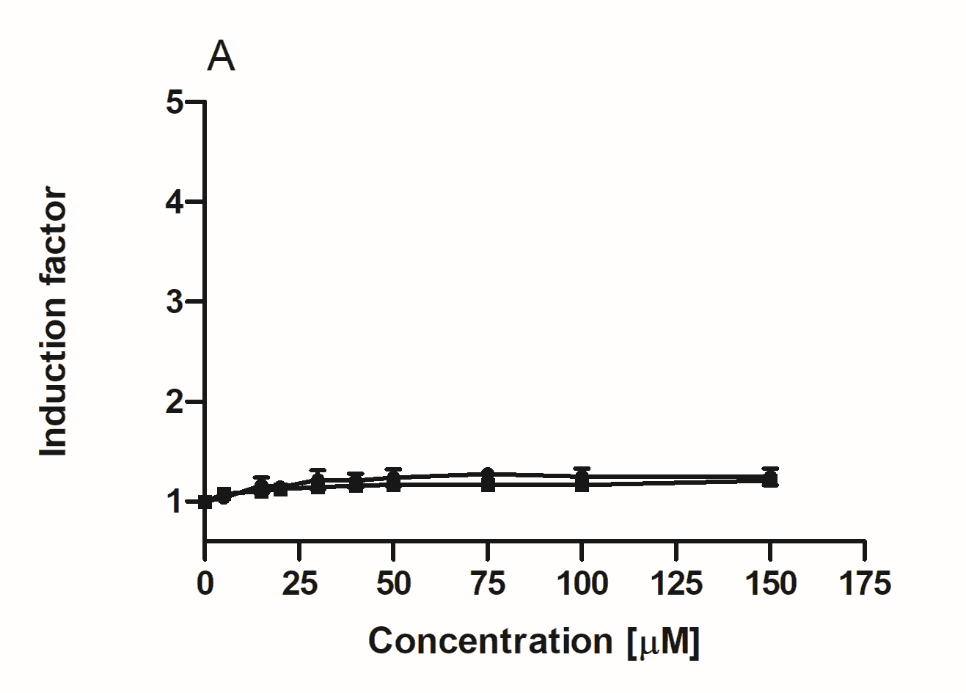

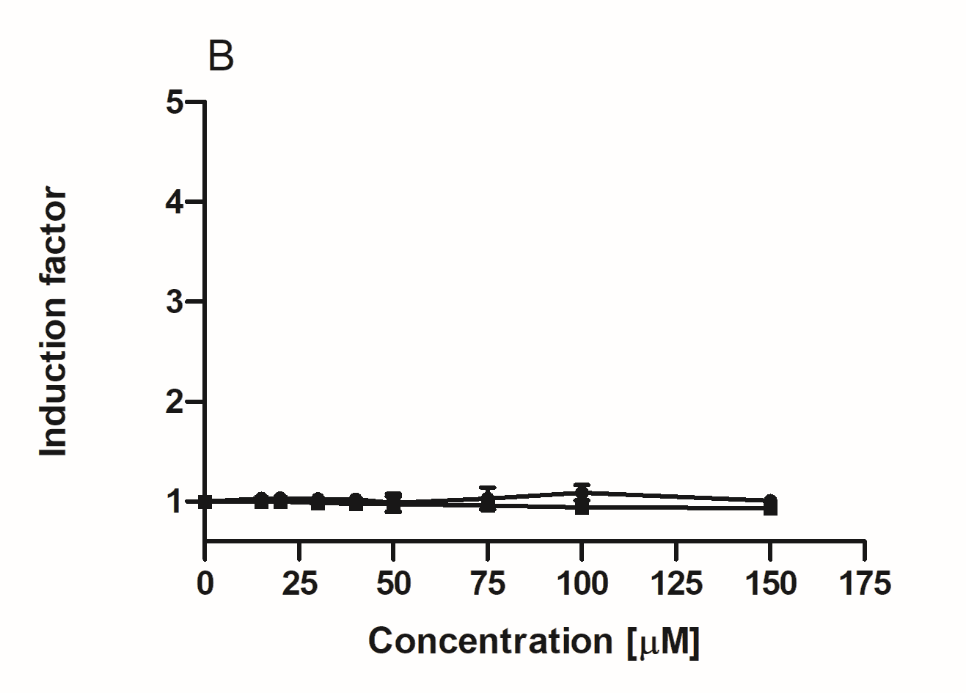
**

**Figure S3.** Induction of luciferase expression in U2OS Cytotox CALUX cells after 24 h exposure to EC (circles) and 3,4-diHPV (squares) with (Figure S2A) and without (Figure S2B) the presence of 0.5 mM L-ascorbic acid in the exposure medium. Results are presented as mean ± SEM compared with the solvent control, derived from three independent experiments.


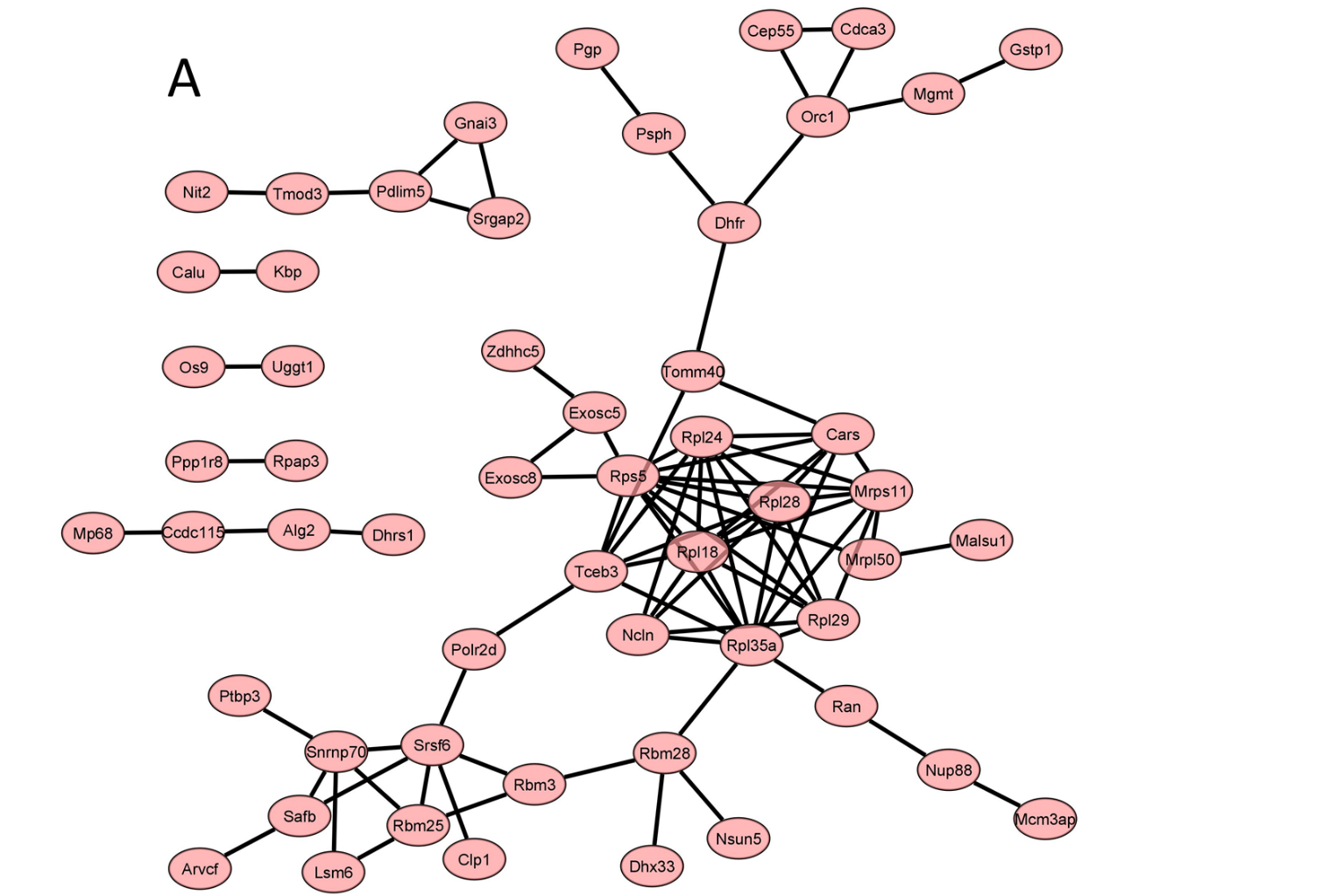


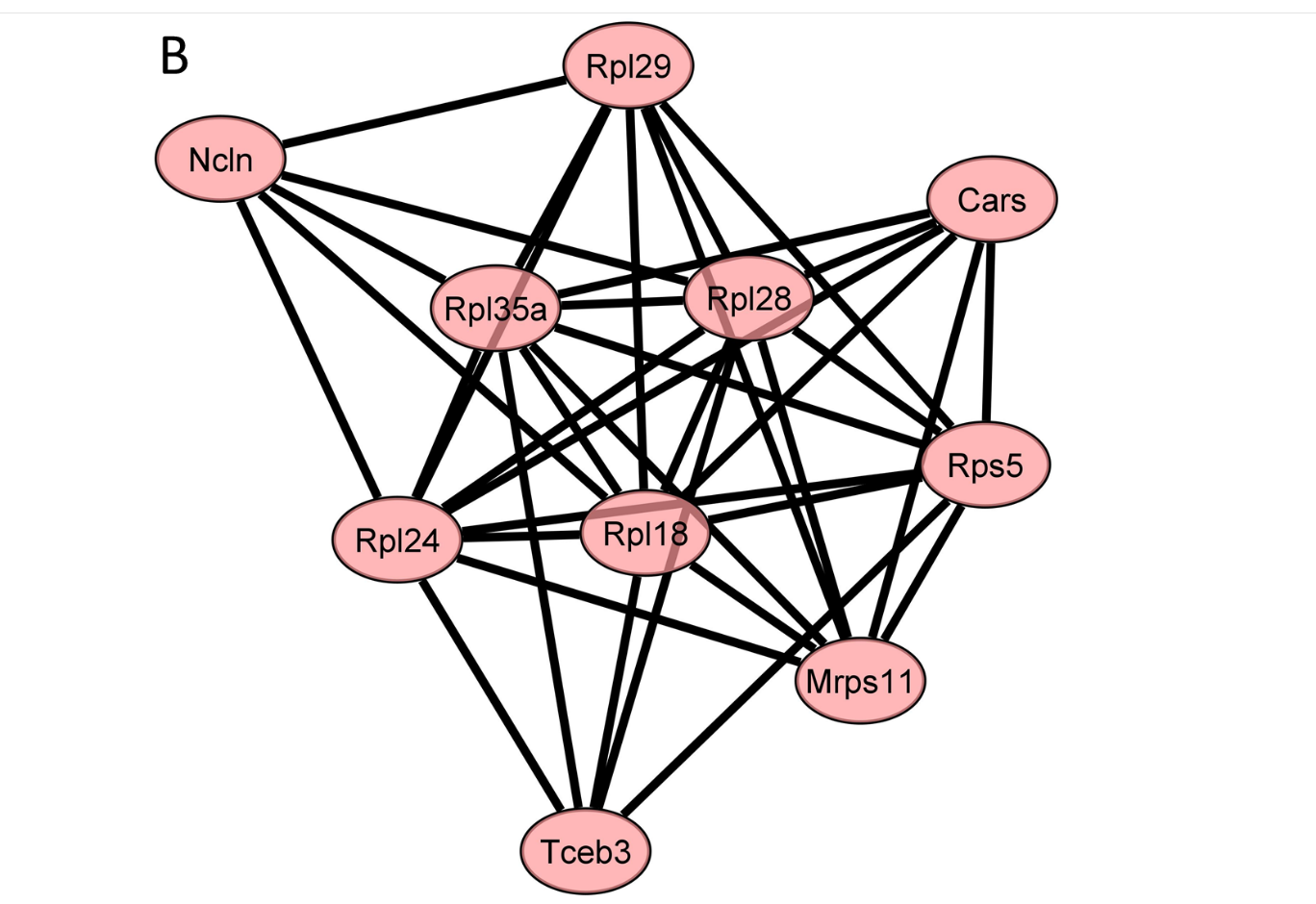


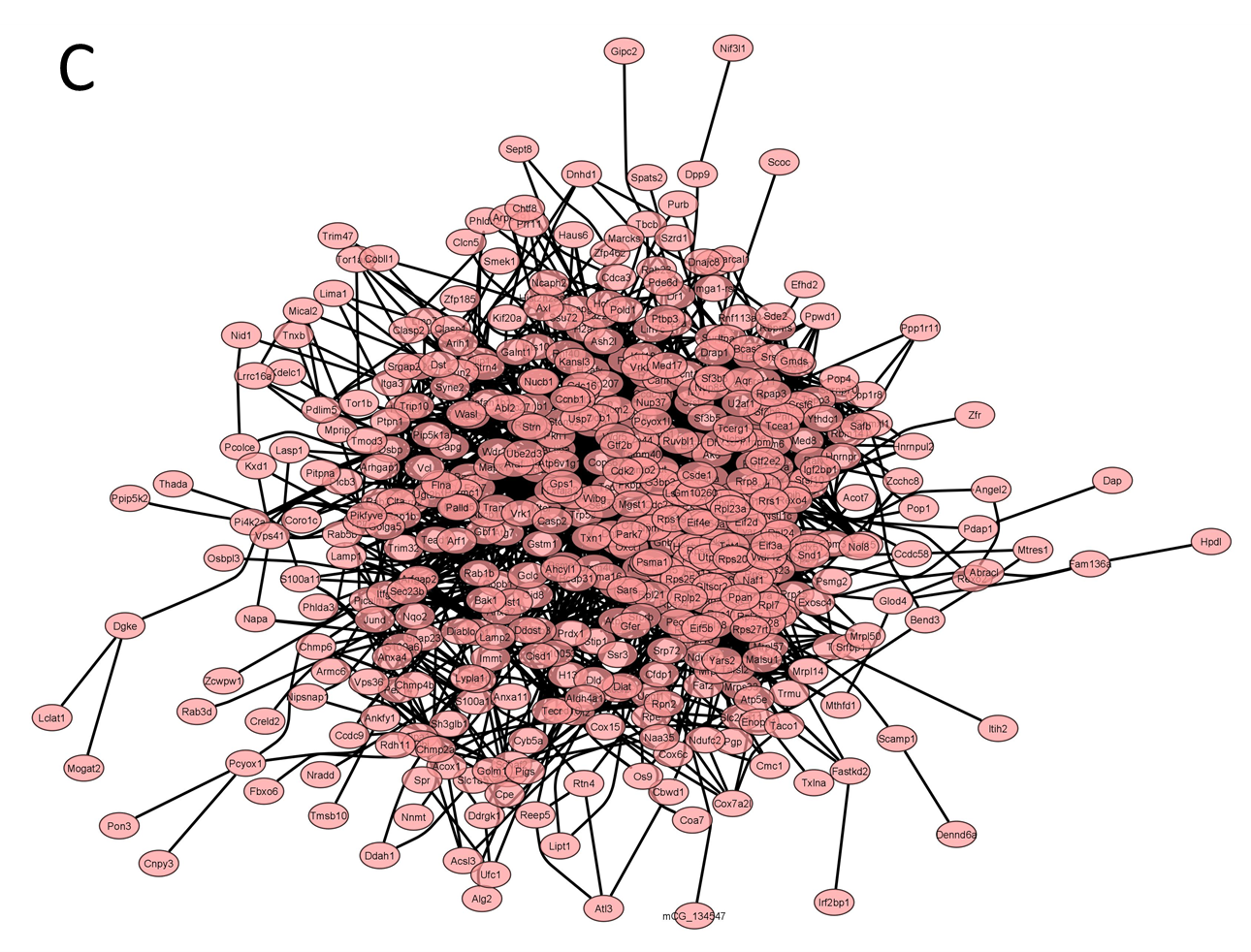


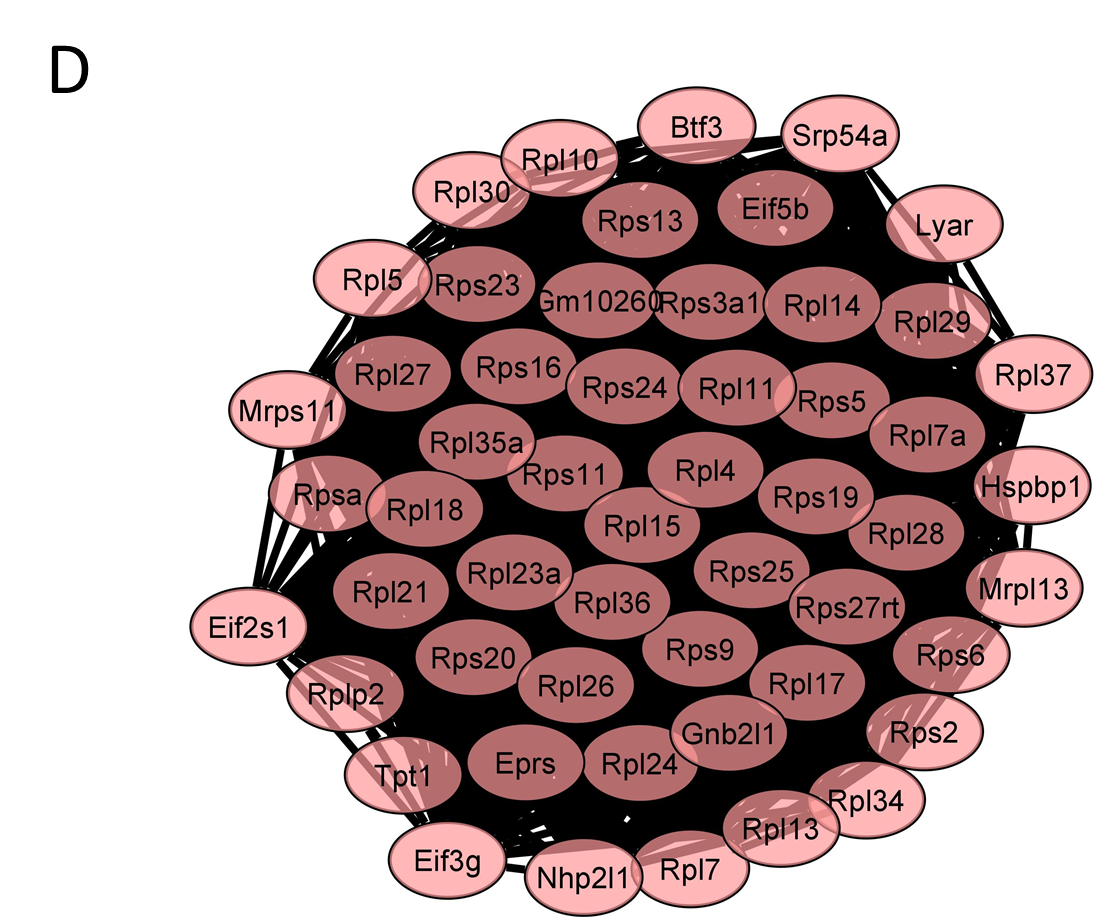


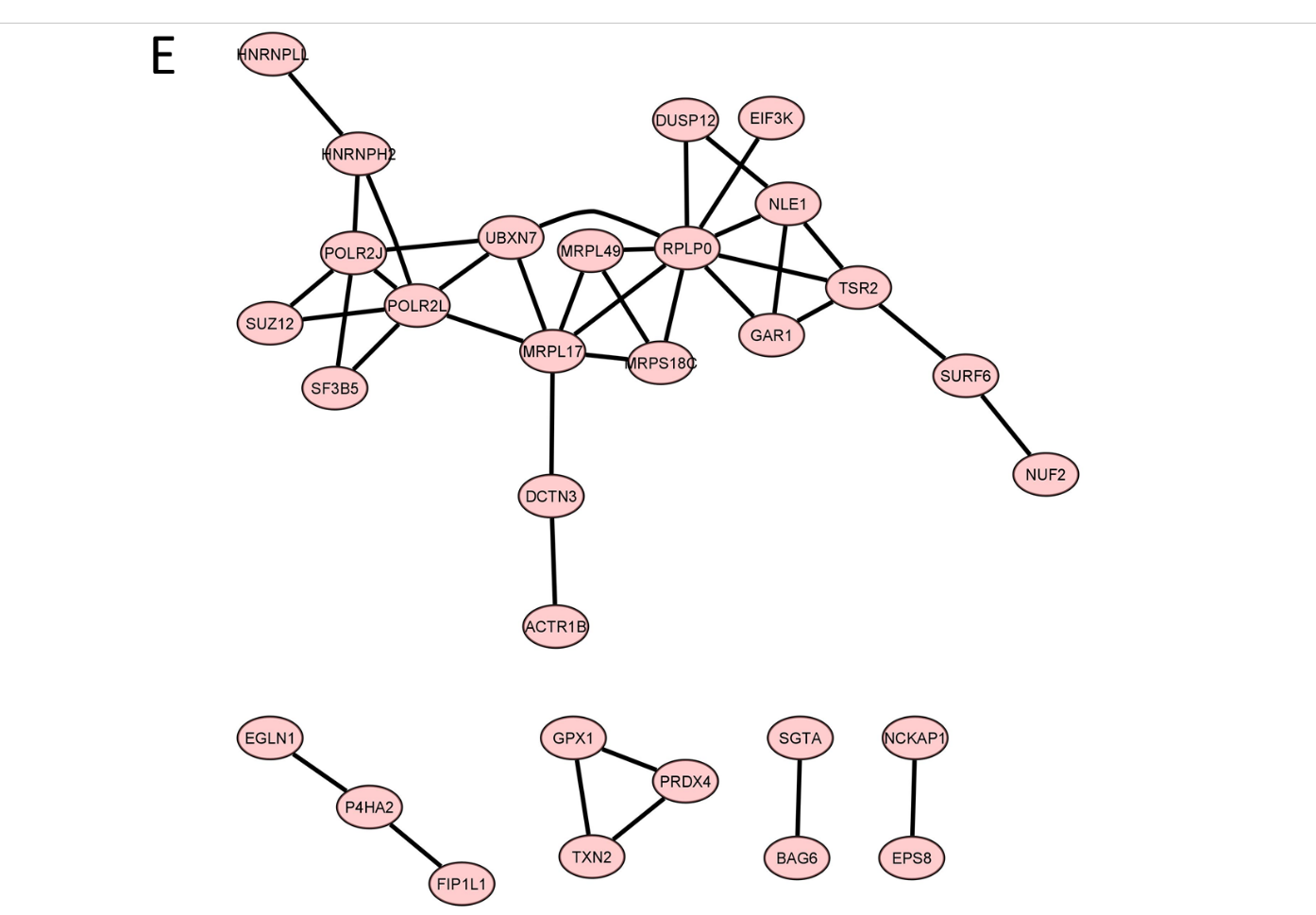


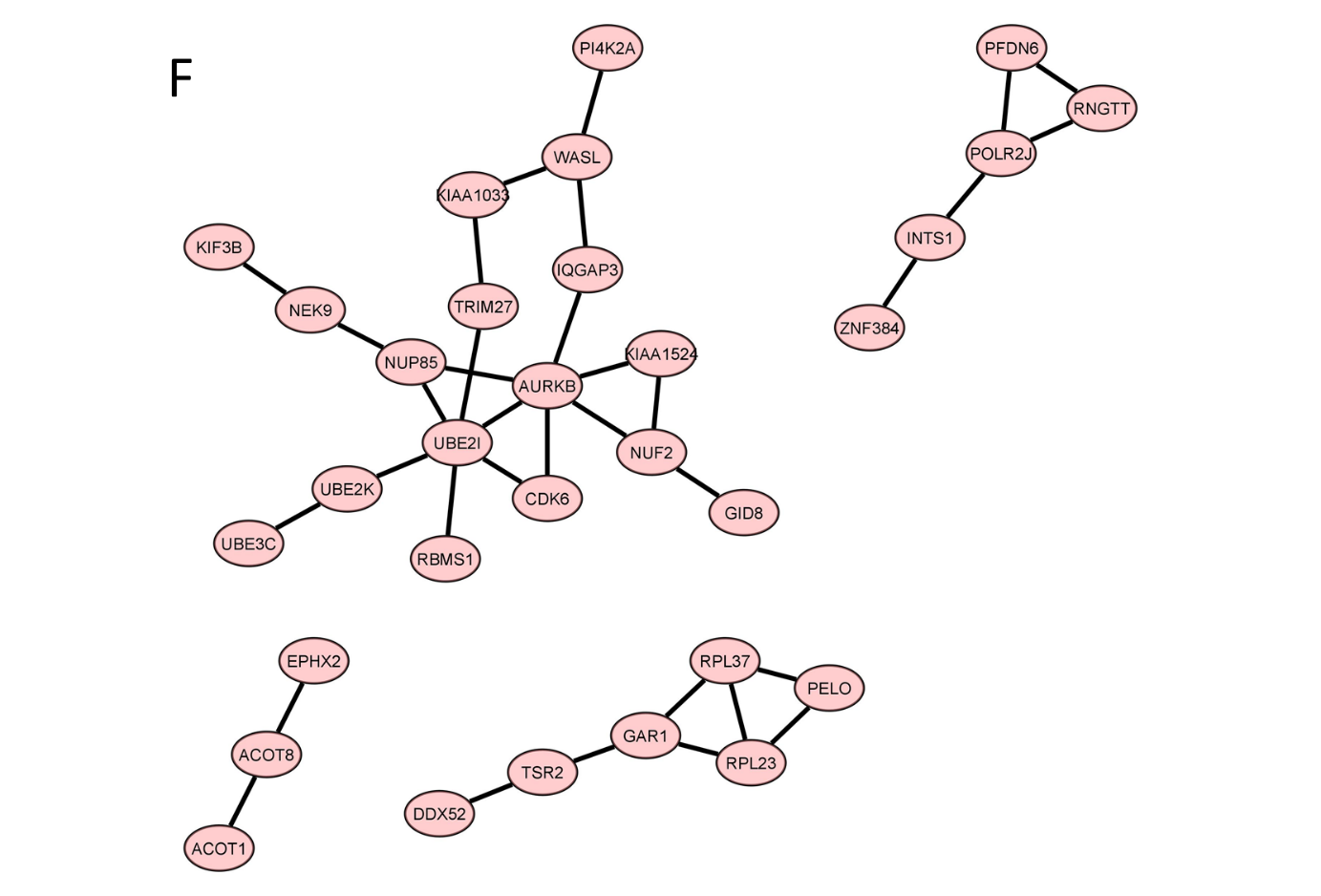


**Figure S4.** Protein-protein interaction (PPI) network analysis of DEPs in Hepa1c1c7 cells and Caco-2 cells after EC and 3,4-diHPV exposure. A and B: PPI network and its hub protein network of DEPs from EC treated Hepa1c1c7 cells. C and D: PPI network and its hub protein network of DEPs from 3,4-diHPV treated Hepa1c1c7 cells. E: PPI network of DEPs from EC treated Caco-2 cells. F: PPI network of DEPs from 3,4-diHPV treated Caco-2 cells.


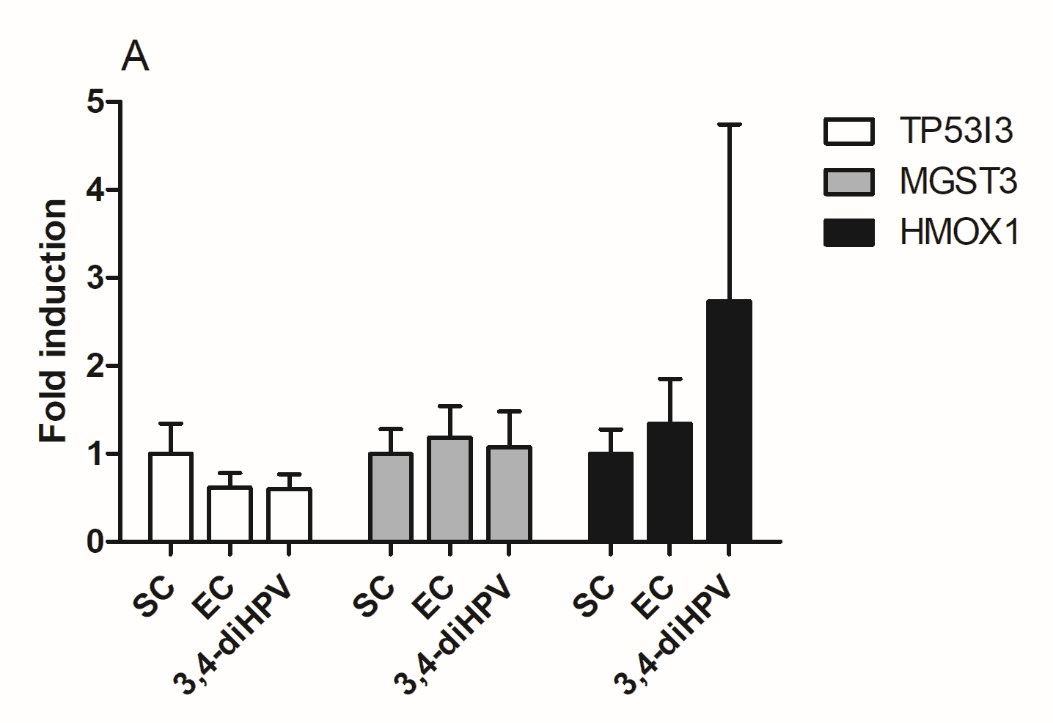

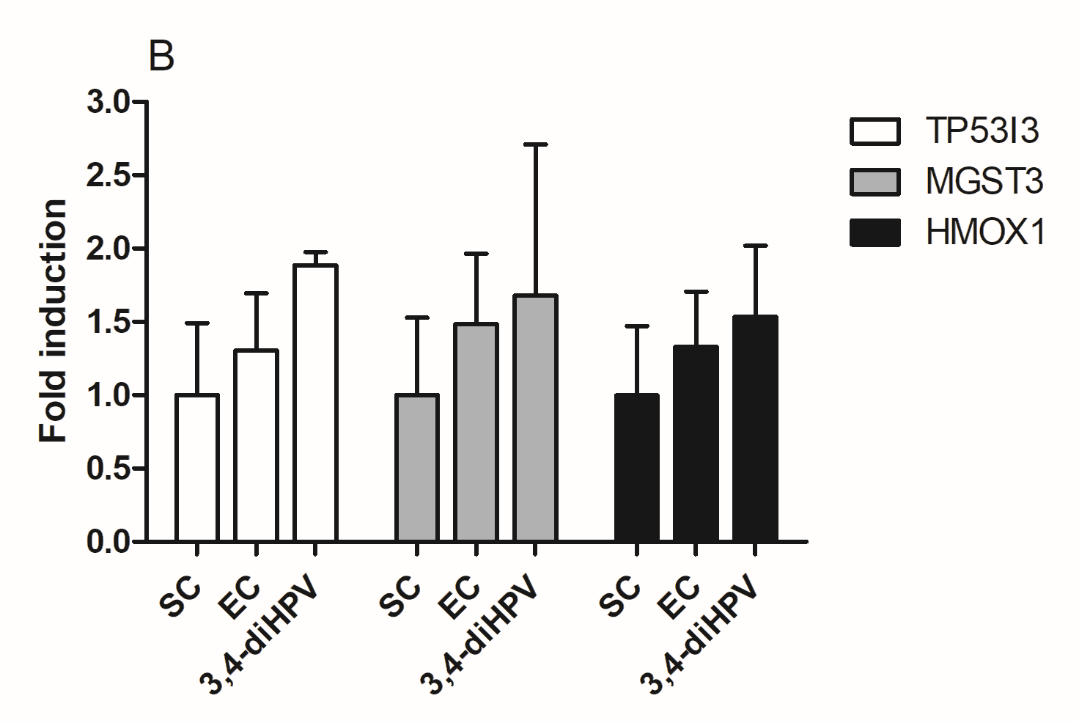


**Figure S5.** Relative RNA (A) and protein (B) levels in Caco-2 cells after exposure to the EC or 3,4-diHPV at 30 µM. Gene/protein names are depicted as figure legends. The results were calculated as the ratio of the treatment and the solvent control (SC) and are presented as mean ± SEM, derived from at least four independent experiments.
